# The acute effects of aerobic exercise on sleep in patients with depression: study protocol for a randomized controlled trial

**DOI:** 10.1186/s13063-019-3415-3

**Published:** 2019-06-13

**Authors:** Gavin Brupbacher, Doris Straus, Hildburg Porschke, Thea Zander-Schellenberg, Markus Gerber, Roland von Känel, Arno Schmidt-Trucksäss

**Affiliations:** 1 Division of Sports and Exercise Medicine, Department of Sport, Exercise and Health, University of Basel, Birsstrasse 320 B, 4052 Basel, Switzerland; 2 OBERWAID AG, Rorschacher Strasse 311, 9016 St. Gallen, Switzerland; 3 Department of Psychology, Division of Clinical Psychology and Epidemiology, University of Basel, Basel, Switzerland; 4 Division of Sport and Psychosocial Health, Department of Sport, Exercise and Health, University of Basel, Birsstrasse 320 B, 4052 Basel, Switzerland; 5 Department of Consultation-Liaison Psychiatry and Psychosomatic Medicine, University Hospital Zurich, Culmannstrasse 8, 8091 Zurich, Switzerland

**Keywords:** Exercise, Depression, Sleep, Polysomnography, Heart rate variability, Blood pressure, Randomized controlled trial, Protocol

## Abstract

**Background:**

Unipolar depression is one of the most important mental disorders. Insomnia is a symptom of cardinal importance in depression. It increases the risk to develop depression, negatively affects disease trajectory, is the most common symptom after remission, increases the risk of relapse, and is associated with higher suicide rates. Existing therapies for insomnia in depression have limitations. Further adjuvant therapies are therefore needed. Acute aerobic exercise has been shown to have beneficial effects on sleep in healthy individuals and patients with insomnia. We therefore hypothesize that a single session of aerobic exercise has a positive impact on sleep in patients with unipolar depression. This trial aims to investigate the effects of a single bout of aerobic exercise on the subsequent night’s sleep in patients with depression.

**Methods/design:**

This is a two-arm parallel group, randomized, outcome assessor blinded, controlled, superiority trial. Patients between 18 and 65 years of age with a primary diagnosis of unipolar depression (without a psychotic episode) are included. Exclusion criteria are regular use of hypnotic agents, opioids, and certain beta-blockers, as well as the presence of factors precluding exercise, history of epilepsy, restless legs syndrome, moderate obstructive sleep apnea, and a BMI > 40. The intervention is a single bout of aerobic exercise, performed for 30 min on a bicycle ergometer at 80% individual anaerobic threshold. The control group sits and reads for 30 min. The primary outcome is sleep efficiency measured by polysomnography. Secondary outcomes include further polysomnographic variables, subjective pre-sleep arousal, nocturnal cardiovascular autonomic modulation, subjective sleep quality, daytime sleepiness, and adverse events. According to the sample size calculation, a total of 92 patients will be randomized using minimization.

**Discussion:**

This trial will add new information to the body of knowledge concerning the treatment of insomnia in patients with depression. Thereby, the results will inform decision makers on the utility of acute aerobic exercise.

**Trial registration:**

Clinicaltrials.gov, NCT03673397. Protocol version 1 registered on 17 September 2018.

**Electronic supplementary material:**

The online version of this article (10.1186/s13063-019-3415-3) contains supplementary material, which is available to authorized users.

## Background

Unipolar depression is a mental disorder of paramount importance. Worldwide the lifetime prevalence is estimated to be between 10 and 15% [1]. It is projected to become the leading cause of burden of disease worldwide by 2030 [2]. Core symptoms of depression are depressed mood, anhedonia, and a lack of drive. Depression is associated with an increased risk of comorbidities [3] and cardiovascular mortality [4]. It is also associated with lower cardiorespiratory fitness [5], an independent risk factor for cardiovascular mortality in healthy individuals [6].

There is a plethora of research concerning treatments for depression. Psychotherapy, pharmacotherapy or a combination of both are the primary treatments for depression according to guidelines [7, 8]. Meta-analytic data show moderate to large effect sizes for pharmacotherapy (0.35), psychotherapy (0.37), and combined therapy (0.74) when compared to placebo [9]. However, the STAR*D trial investigating four treatment steps in over 4000 patients concluded that only two-thirds of people treated for depression with pharmacotherapy, cognitive behavioral therapy, or both were in remission after treatment [10]. Moreover, the majority of patients tend to prefer non-pharmacological treatments for insomnia [11, 12] and depression [13]. Hence, there is a need for further adjuvant non-pharmacological treatment options.

Insomnia encompasses problems initiating or maintaining sleep with daytime impairments [14]. Another symptom may be non-restorative sleep. The current state of knowledge suggests that hyperarousal [15, 16], as well as sleep reactivity [17], are core etiological factors of insomnia. Hyperarousal can be cognitive (e.g., rumination, dysfunctional beliefs), emotional (emotional reactivity), cortical (beta activity in sleep EEG), or physiological (e.g., metabolic rate, heart rate variability).

There is an abundance of research related to insomnia treatments. Cognitive behavioral therapy for insomnia (CBT-I) and pharmacotherapy are currently considered first- and second-line therapies for insomnia (regardless whether comorbidities are present or not) [17, 18]. CBT-I is very effective [19]. However, the number of trained specialists considerably limits access to this treatment. Pharmacologic agents such as benzodiazepines, non-benzodiazepines, and sedating antidepressants are another, arguably more frequently administered therapy for insomnia. However, pharmacotherapy has multiple limitations: (1) effects have been shown to be statistically significant but of limited clinical relevance [20, 21], (2) dosages and (3) prescription duration frequently exceed recommendations of health agencies [22, 23] (especially in patients with comorbidities [24, 25]), (4) it potentially has severe adverse effects [26–28], and (5) patients often prefer non-pharmacological therapies [11, 29, 30]. Therefore, there is a need to develop further non-pharmacological treatments.

Insomnia is a highly relevant symptom of depression. Depending on the methodological approach, studies have found 25–90% prevalence rates of insomnia in people with depression [31, 32]. Longitudinal studies have repeatedly found a bidirectional link between insomnia and depression [33–36]. Insomnia is of prognostic relevance. It negatively affects the disease trajectory [37], is the most frequent residual symptom after treatment response or remission [31, 38, 39], increases the probability of relapse [37, 40, 41], and is an independent risk factor for suicide [42, 43] as well as adverse somatic outcomes, particularly cardiovascular disease [44]. Insomnia has considerable economic cost. Individuals who have insomnia are at higher risk for work presenteeism [45, 46] and absenteeism [47] with higher direct costs per short-term absence [48]. Sleep problems are also associated with more work injuries [49]. Insomnia increases the risk of ending employment prematurely [50] and increases the risk of disability retirement due to depression [51].

Insomnia of depressed individuals has been neglected in research, despite its known relevance. Until approximately one decade ago, a central etiological distinction was made between ‘organic’ and ‘psychogenic’ or ‘primary’ and ‘secondary’ insomnia [52]. However, this distinction has been challenged because, among other reasons, there is often a lack of evidence for a mechanistic distinction between primary and secondary insomnia [52]. Therefore, a paradigm shift has become apparent, recommending specific treatments for comorbid insomnia. This paradigm shift might explain why, until recently, most trials have focused on patients with insomnia without any comorbidities.

Aerobic exercise is a viable candidate for the treatment of insomnia in patients for depression. Meta-analyses have shown a positive impact of acute and chronic exercise in healthy individuals with small to moderate effect sizes [53, 54]. Meta-analyses focusing on individuals with at least mild insomnia but no comorbidities have found a moderate effect of chronic aerobic exercise on sleep quality [54–56]. Aerobic exercise has further positive effects such as improving depressive symptoms [57] and cardiorespiratory fitness [58]. The latter is especially relevant for the reduction of cardiovascular risk [6].

Current sleep hygiene recommendations, which are also relevant for depression, lack feasibility. In particular, they state that exercise should not be performed after 2 pm [59]. This time constraint presents a considerable limitation since many people can only accommodate aerobic exercise in the late afternoon or evening. Even more so this may be limiting for patients with morning depression who may feel more energetic to exercise in the afternoon [60]. However, a recent meta-analysis has found equivocal effects of exercise performed in the afternoon or evening in healthy individuals [54]. In healthy individuals, effects of a single evening bout of aerobic exercise on nocturnal heart rate variability seem to depend on intensity, duration, and timing relative to sleep but do not seem to alter subjective sleep quality [61, 62]. One trial has investigated the effects of acute aerobic exercise in chronic primary insomniacs, showing moderate to large effect sizes for shortened sleep onset latency, improved sleep efficiency, and longer total sleep time [63].

### Rationale and hypotheses

An extensive literature search yielded no randomized controlled trials investigating the acute effects of exercise on sleep in patients with depression. Several studies concerned with chronic effects are available, and we will summarize these in our upcoming systematic review and network meta-analysis (PROSPERO ID 115705, registration not published yet). Considering this gap in the literature and the uncertainty concerning the effects of exercise performed in the afternoon on sleep in patients with depression, a trial on this topic is of high clinical importance.

We hypothesize that an acute bout of aerobic exercise improves 1) sleep efficiency [54, 63], 2) sleep continuity [63], 3) sleep architecture [54], 4) subjective sleep quality [64], 5) daytime sleepiness [65], 6) nocturnal blood pressure [66], 7) pre-sleep arousal, and 8) pre- and post-sleep heart-rate variability. We expect no effect on 9) nocturnal heart rate variability [62] and 10) the frequency and severity of adverse events [64].

This paper presents the design and protocol for the trial according to the Standard Protocol Items: Recommendations for Interventional Trials (SPIRIT) statement [67] (Additional file 1).

## Methods/design

### Study design and setting

**Fig. 1:**
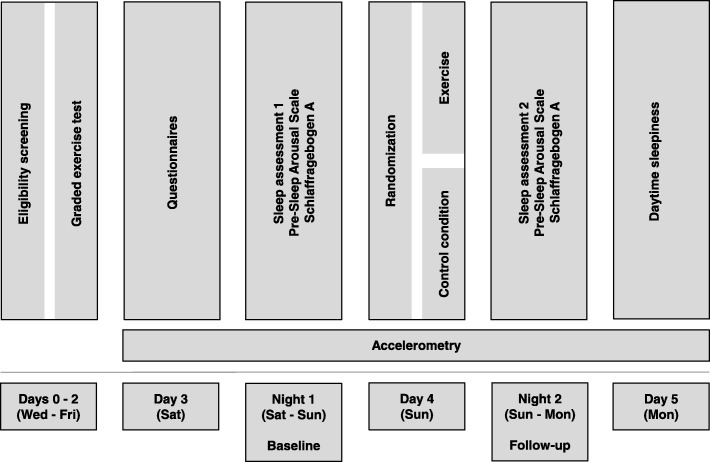
Trial design

This study is designed as a two-arm parallel group, randomized, outcome assessor blinded, controlled trial, to assess the superiority of (i) an acute bout of aerobic exercise compared to (ii) control in ameliorating sleep efficiency in patients with depression (Fig. 1).

The trial is conducted in the psychosomatic inpatient rehabilitation unit of the OBERWAID AG, a rehabilitation clinic in St. Gallen, Switzerland. Patients are referred to the clinic by their general practitioner or a psychiatrist. On average, approximately 250 patients with a primary diagnosis of an ICD-10 depressive episode without psychotic features are referred to the clinic annually. The OBERWAID AG also offers outpatient psychosomatic care, cardiovascular inpatient rehabilitation, and orthopedic aftercare.

### Participants

#### Eligibility criteria

Patients aged 18–65 years with a diagnosis of depression (confirmed by experienced psychiatrists according to ICD-10) undergoing inpatient psychosomatic rehabilitation in the OBERWAID clinic are eligible for inclusion. Further inclusion and exclusion criteria with corresponding rationales are listed in Table 1.

**Table 1:** Inclusion and exclusion criteria

Criterion	Rationale
**Inclusion criteria**	
≥ 18 and ≤ 65 years old	Results of the trial should be generalizable to the working age population. Furthermore, there can be numerous reasons for sleep disorders in older patients [68]
Primary diagnosis of depression (F32, F33) without psychotic episode according to ICD-10	Mental disorder used to define the sample
**Exclusion criteria**	
Regular use of hypnotic agents* (patients are included if no hypnotic agents were taken two weeks before study participation)	Use of hypnotic agents might mask the effect of aerobic exercise on sleep
Factors precluding exercise testing or training	For safety reasons, patients who have any condition which precludes exercise testing or training are excluded. Absolute and relative contraindications are based on ACSM’s Guidelines for Exercise Testing and Prescription [69]
Use of beta-blockers (except carvedilol and nebivolol)	Except for carvedilol and nebivolol [70, 71], beta-blockers have been shown to reduce nocturnal melatonin levels [70, 72, 73]
Use of opioids	Opioids affect sleep architecture [74]
History of epilepsy	Epilepsy is associated with quantitative and qualitative alterations of sleep [75] and might, therefore, mask the intervention effects
Restless legs syndrome defined by ≥ 7 points on the restless legs screening questionnaire [76]	Can also cause sleep disturbance, but the etiology is distinct from depression [77]
Moderate or severe sleep apnea defined by an oxygen desaturation index (ODI) ≥ 15 in the first polysomnography	Sleep apnea is a distinct sleep disorder with clearly delineated etiology. ODI has been shown to be highly correlated with the apnea-hypopnea index and to detect sleep apnea with high sensitivity and specificity [78–80]
Morbid adiposity with BMI > 40	These patients might suffer from hypoventilation syndrome which affects sleep

*ICD-10* International Classification of Diseases, version 10, *ODI* oxygen desaturation index, *BMI* body mass index

*Hypnotic agents are defined as follows: orexin receptor agonists, benzodiazepine receptor agonists, sedating antidepressants, neuroleptics, benzodiazepines, melatonin agonists, heterocyclics, anticonvulsants, over the counter sleep aids (sedating antihistamines, melatonin L-tryptophan, valerian), and cannabinoids

#### Recruitment

Patients are given the study information at the preliminary medical consultation, which takes place approximately 3 weeks (but no later than 2 days) before admission. On the day of admission, the clinical trial is presented to the patients by the study coordinator. Patients who are interested in participating in the study can ask the study coordinator questions directly. In order to ensure adequate time to consider their participation, interested and potentially eligible patients meet the study coordinator for an informed discussion again on the following day (i.e., the first day after admission). The study coordinator obtains the written informed consent from patients willing to participate. Should the study coordinator be absent, his designated replacement obtains the informed consent. The information sheet and consent form are in German (Additional file 2). It is clinical routine for the leading physicians to give all patients a general consent form.

#### Retention

Appointments for data collection are included in the therapy schedule during the first 6 days of rehabilitation, which should reduce the burden on patients and ensure protocol fidelity. We give patients feedback on their measurements. We will send a digital copy of publications based on this trial to participants if they have chosen to receive one. No monetary compensation is offered for participation.

#### Withdrawal, discontinuation, and stopping rules

Participants can withdraw from the trial at any time without having to provide reasons. If possible, we collect follow-up data and reasons for withdrawal. Patients can be withdrawn from the trial by the principal investigator for medical reasons (e.g., transfer to another hospital). Exercise testing and training is immediately stopped, should any indications for exercise test termination, as defined by the American College of Sports Medicine, be met [69]. In this case, we collect follow-up data if there are no contraindications.

### Measurements and procedure

#### Rationale for measurements

This section offers a rationale for the selected variables and performed measurements. Details of each assessment (measurement variable, reliability, validity, analysis metric, method of aggregation, time point, and clinical relevance) are provided below.

##### Baseline

**Fig. 2:**
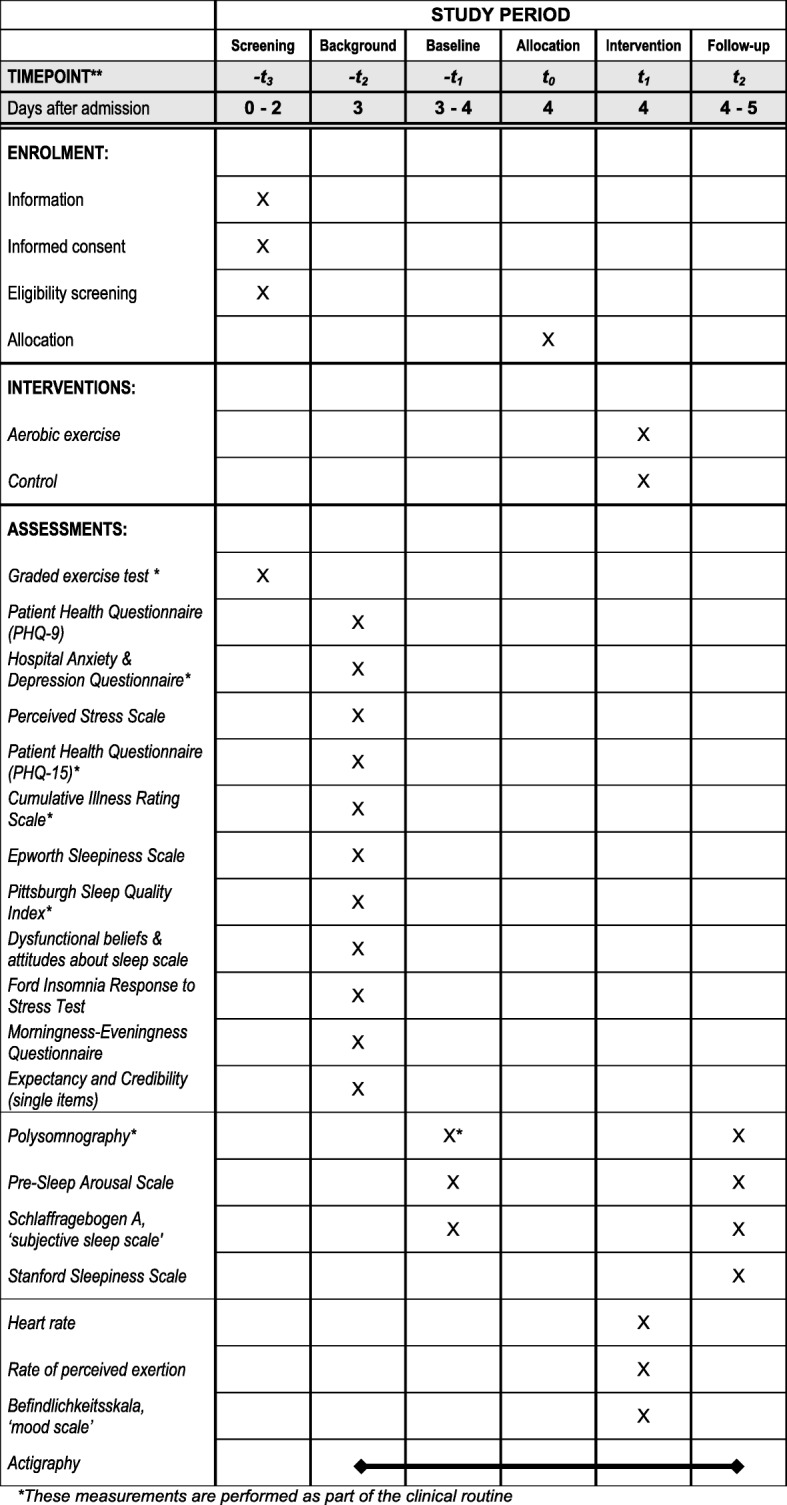
Participant timeline - Spirit figure

This trial does not exclude patients with psychiatric comorbidities in order to preserve external validity. Therefore, we perform an extensive baseline characterization of the sample (Fig. 2). The characterization consists of questionnaires on psychiatric, somatic, and insomnia-specific symptom severity as well as sleep-related variables such as chronotype and sleep-related cognitions. Furthermore, baseline polysomnography will provide an extensive characterization of sleep.

##### Primary outcome

Sleep disorders are of complex etiology and have various psycho-physiological consequences. Therefore, it is recommended to measure the effects of interventions on insomnia symptoms as well as other factors such as daytime functioning and mood [81]. Insomnia can be classified into sleep-onset, sleep-maintenance, and sleep-offset subtypes. Meta-analysis of polysomnographic data has shown that patients with depression and other mental health disorders have mixed (i.e., sleep-onset and sleep-maintenance) insomnia [82]. Sleep efficiency best captures both of these aspects and is therefore chosen as the primary outcome. Objective quantification of the primary outcome is vital in this study since the blinding of participants is not possible, thereby potentially affecting subjective measurements.

##### Secondary outcomes

Secondary outcomes which inform clinical decision making include further polysomnographic variables, subjective pre-sleep arousal, cardiovascular autonomic modulation, subjective sleep quality, daytime sleepiness, and adverse events. Wake after sleep onset and number of awakenings identify sleep-maintenance insomnia, while sleep-onset latency characterizes sleep-onset insomnia.

Patients with depression show characteristic changes in rapid eye movement (REM) latency (time between sleep onset and first rapid eye movement sleep episode), REM-density (frequency of rapid eye movements/REM episode), and duration of REM sleep (collectively known as REM pressure) [82]. More importantly, these alterations have a negative impact on treatment and increase the risk of relapse [83]. Exercise has been shown to reduce REM sleep and increase slow wave sleep [54]. The discrepancy between objective and subjective sleep measurements is well documented [81, 84, 85]. Therefore, we measure the subjective sleep quality of the previous night in addition to the objective sleep assessment. Patient views influence the treatment of sleep disorders [86, 87]. Hence, it is crucial to ascertain the credibility of the intervention and the expectancy of participants. Adverse events are underreported in sleep trials but highly relevant to clinical decision making [88]. The case report form specifically captures adverse events (see below) to gauge the benefit to harm ratio.

#### Screening

After providing informed consent, we formally screen patients for inclusion and exclusion criteria of this study. They are consulted by an experienced psychiatrist and undergo a full history and medical examination by an experienced internist, including vital parameters. (Resting electrocardiogram may be forgone if the general practitioner of the patient provides one no older than 2 months.) Patients with undiagnosed sleep apnea are excluded according to the baseline polysomnography (see below).

#### Graded exercise testing

Patients fulfilling all eligibility criteria (except the sleep apnea criterion, which is determined later by polysomnography) undergo sub-maximal graded exercise testing on a bicycle ergometer (ergoselect 200, Ergoline, Bitz, Germany). The goal is to determine the individual anaerobic threshold. The anaerobic threshold is used to standardize the exercise intensity of the intervention across patients. Since subjects vary in their endurance capacity and weight, we adjust initial and subsequent work rates (Watts) between subjects before the test. Stage duration is always 3 minutes, as this has been found to yield the most reliable and valid results [89]. We measure blood lactate at the end of each stage using capillary blood from the earlobe. We assess blood lactate level with Lactate Scout+ (SensLab GmbH, Leipzig, Germany), a validated hand-held analyzer [90]. Heart rate is measured using the validated Polar® H7 chest strap (Polar OY, Finland) [91]. Parasympathetic drive is still present at lower exercise intensities and thus increases heart rate variability [92]. The average heart rate during the last 30 s of each stage is extracted to improve precision and used for further analyses. Ratings of perceived exertion are recorded at the end of each stage according to Borg’s 6–20 scale [93]. The graded exercise test data are analyzed using a specialized software program (Ergonizer, Freiburg, Germany). The individual anaerobic threshold is determined according to the method of Dickhuth et al. [94].

#### Baseline characterization of symptom severity

To characterize the study population in detail, we will report multiple continuous measures at baseline as median with corresponding interquartile range or frequencies.

##### Somatic and psychological symptom severity

In accordance with the International Consortium for Health Outcomes Measurement (ICHOM) Depression and Anxiety working group, depression symptoms are assessed with the German version of the Patient Health Questionnaire-9 (PHQ-9) including the additional question on functioning [95]. The nine symptom items are scored on a four-point Likert scale (*not at all* to *nearly every day*; Cronbach’s α = 0.89). The cut-offs of the aggregated sum score have been validated and allow for classification of mild to severe depression [96]. Anxiety is assessed using the Hospital Anxiety and Depression Scale. This questionnaire measures depression and anxiety with seven items each on a four-point Likert scale [97]. A meta-analysis has found Cronbach’s α to be 0.83 and 0.82 for the anxiety and depression subscales, respectively [98]. Diagnostic test accuracy of the depression and anxiety subscale is high [99]. More recently, a meta confirmatory factor analysis has suggested the presence of a general distress factor explaining most of the covariance between items [100]. The German version has been shown to have adequate psychometric properties [101]. We measure stress with the German version of the ten-item Perceived Stress Scale, which has demonstrated good reliability (Cronbach’s α = 0.84) and validity [102]. Therein, stress is operationalized as the degree to which life is experienced as unpredictable, uncontrollable, and overloaded in the past months on a five-point Likert scale (*never* to *very often*). Somatic multimorbidity is measured using the self-administered Patient Health Questionnaire Somatic Symptom Scale (PHQ-15), which has good reliability (Cronbach’s α = 0.8) and validity [103]. This questionnaire measures the presence and severity of somatic symptoms during the past 4 weeks on a three-point Likert scale (*not bothered at all* to *bothered a lot*). The items assess symptoms clusters which account for more than 90% of physical complaints reported in out-patient settings [103]. Lastly, the German version of the Modified Cumulative Illness Rating Scale provides physician-rated scores of multimorbidity. This scale has a good inter-rater agreement and concurrent and predictive validity. It measures the presence and severity multimorbidity over 14 organ systems on a five-point scale (*no problem* to *extremely severe problem*) [104].

##### Subjective measurements related to sleep

Multiple sleep-related variables are measured to characterize the impact of sleep disorders in the population. The German version of the Pittsburgh Sleep Quality Index [105] is used to quantify subjective sleep disturbance. This 18 item scale assesses subjective sleep quality, sleep latency, sleep duration, habitual sleep efficiency, sleep disturbances, use of sleeping medication, and daytime dysfunction. It has adequate psychometric properties (Cronbach’s α = 0.83) [106]. The corresponding global score with a cut-off value of ≥ 5 has been shown to distinguish good and poor sleepers [107]. We measure sleep reactivity, i.e., the degree to which an individual experiences disrupted sleep due to stress, with the Ford Insomnia Response to Stress Test [108, 109]. The nine-item self-report questionnaire is answered on a four-point Likert scale (*not very likely* to *very likely*) with higher total scores indicating a higher likelihood of stress-induced insomnia. The reliability (Cronbach’s α = 0.80) and validity of the German version have been demonstrated [110]. Dysfunctional sleep-related thoughts and attitudes are related to the development and trajectory of sleep disorders [111]. The reliability and validity of the German short form of the dysfunctional beliefs and attitudes about sleep scale have recently been demonstrated (average Cronbach’s α = 0.71) [112]. Sixteen items measure four domains (consequences, worry/helplessness, expectations, medication) on a ten-point Likert scale (*strongly disagree* to *strongly agree*). Excessive daytime sleepiness is associated with insomnia and depression [113, 114] as well as reduced quality of life and work performance [115]. We assess chronic daytime sleepiness using the German version of the Epworth Sleepiness Scale. This questionnaire has good reliability (Cronbach’s α 0.83 in patients) and validity [116]. The likelihood of dozing off in eight daily situations (e.g., sitting and reading) is assessed on a four-point Likert scale (*would never doze* to *high chance of dozing*). Chronotype, i.e., peak alertness throughout the day, is associated with more severe depression, insomnia, and suicidality in patients with major depression. Furthermore, eveningness substantially increased the risk of non-remission independently of insomnia severity [117]. The Morningness-Eveningness Questionnaire assesses chronotype with 19 multiple-choice questions (four- or five-point scale) on sleep habits and propensity for performance throughout the day. The sum ranges from 16 to 86 and can be translated into chronotypes (< 42, evening type; 42–58, neither; > 58, morning type) [118]. Criterion validity has been established [119]. Validity and reliability (test-retest reliability > 0.96) have been confirmed for the German version [120].

#### Polysomnography (baseline and follow-up)

Polysomnography is considered the gold standard of sleep assessment [121]. However, one significant drawback of this method is the so-called *first-night effect*. This effect describes altered sleep patterns due to novel environments, disturbances by measurement equipment, a potential Hawthorne effect, or a combination thereof [122]. Such alterations typically represent worse sleep quality, e.g., reduced sleep efficiency, in the first compared to the second night [122]. Although this effect has been found in different patient groups, it is clearly attenuated in individuals with depression [123–125]. Moreover, polysomnographic measurements are expensive and performing multiple measurements is not always feasible. Several authors argue that data from the first night (i.e., baseline) should, therefore, be used in the analyses [123, 125]. In designs where baseline and follow-up data are collected, analysis of covariance (ANCOVA) has multiple advantages. Firstly, there is less potential for bias compared to the analysis of change (i.e., pre minus post) scores or follow-up data only. Secondly, ANCOVA has higher statistical power [126, 127].

##### Montage

Polysomnography is performed with the SOMNOscreen™ plus RC (Somnomedics, Randersacker, Germany) using the following montage: one EEG channel (Fp2-A1, 512 Hz), two EOG channels (1 cm below and 1 cm lateral of the outer right canthus as well as 1 cm above and 1 cm lateral of the outer left outer canthus, 512 Hz), one EMG channel (Chin1-Chin2, 512 Hz), one ECG channel (modified lead II, 512 Hz), thoracic respiratory effort channel (inductance plethysmography belt, 32 Hz), finger photoplethysmography (non-dominant arm, 128 Hz), body position (stored every 30 s), movement (32 Hz), and ambient light (stored every 30 s). Relevant skin areas are prepared with Nuprep (Weaver & Co., Aurora, CO, USA) before electrode placement to reduce skin impedance. Although this montage does not comply with AASM standards [128], the use of a single channel EEG, two EOG channels, and one EMG channel has been shown to have good validity when assessing sleep stages [129].

##### Analysis of polysomnography

We apply low- and high-frequency filters according to AASM guidelines [128]. Oxygen desaturation index (ODI) is defined as the number of oxygen desaturations (≥ 4%) measured by photoplethysmography per hour of total sleep time [128]. We analyze polysomnography data visually in epochs of 30 s by two trained, independent, and blinded raters according to AASM guidelines [128]. If sleep efficiency differs by ≤ 5% between raters, we use the mean of each quantitative sleep parameter of both raters for analysis. If sleep efficiency differs by > 5% between raters, a consensus scoring is done by a third rater who is blinded against allocation and previous ratings. Inter-rater reliability (intraclass correlation) based on the scoring of the first two raters will be provided for the primary outcome. Sleep parameters are calculated with the proprietary DOMINO software (Somnomedics, Randersacker, Germany) (Table 2).

**Table 2:** Sleep parameters

Sleep parameter	Definition
Total recording time (TRT)	Time between the lights-off and lights-on markers (min)
Total sleep time (TST)	Time asleep (in any sleep stage) within TRT (min) TST = N1 + N2 + N3 + REM
Sleep onset latency (SOL)	Time between lights-off marker and first epoch of any sleep stage
Wake after sleep onset (WASO)	Time awake after first sleep episode (min) WASO = TRT - SL - TST
Number of awakenings (NA)	Number of wake periods of at least two epochs after sleep onset
Sleep efficiency (SE)	Percentage of sleep while in bed (%) SE = (TST / TRT) × 100
N1	Stage 1 (in minutes and % TST)
N2	Stage 2 (in minutes and % TST)
N3	Stage 3 (in minutes and % TST)
Light sleep	Stage 1 and 2 (in minutes and % TST)
NREM (non-REM) sleep	Stage 1–3 (in minutes and % TST)
REM	Rapid eye movement (in minutes and % TST)
REMLAT	Time between sleep onset and the occurrence of the first REM sleep epoch (min)
Stage shift index	Number of transitions between any wake or sleep stage/hours of sleep

Sleep parameters can be grouped into three domains:

1. Sleep continuity: high sleep efficiency, low sleep onset latency, low wake after onset, low number of awakenings
2. Sleep depth: less stage 1 and 2 sleep, more stage 3 (i.e., slow wave) sleep
3. REM pressure: low REM latency, more REM sleep, (high REM-density)

##### *Nocturnal* autonomic cardiovascular modulation

Sleep hygiene recommendations include daily exercise. Two caveats of this recommendation are that exercise should be performed before 2 pm and that no strenuous exercise ought to be done close to bedtime. One reason for this recommendation is that exercise in the afternoon or evening might increase arousal and thereby prevent sleep [59]. However, evidence from numerous epidemiological, observational, and experimental studies have repeatedly failed to show such an adverse effect or have found that the opposite is true [54, 130]. The issue of nocturnal autonomic modulation following exercise is of central importance for three reasons. Firstly, arousal is integral to one of the most widely accepted etiological theories of insomnia [15, 16]. Secondly, heart rate variability (HRV), as a marker of autonomic arousal, has been proposed as a potential pathophysiological mechanism linking depressive disorders [131, 132] and insomnia [133] with cardiovascular disease. Thirdly, nocturnal blood pressure and nocturnal blood pressure dipping are of prognostic relevance for cardiovascular disease [134]. Therefore, sleep quality, as well as nocturnal autonomic activity, should be assessed when evaluating interventions in sleep research. Measurements of heart rate, HRV, and pulse transit time (and thereby calculated blood pressure) are collected to quantify the effect of exercise on parameters of autonomic cardiovascular arousal.

We record the ECG while subjects are lying in bed. Patients were instructed to lie in the supine position and refrain from speaking as well as moving during the two short-term (i.e., 5-minute) recordings. No instructions concerning breathing were given. All ECG measurements were sampled at 512 Hz using modified lead II.

Three sections of HRV data are analyzed:

1. A short-term pre-sleep measurement with a duration of 5 min, beginning after lights off, is taken to quantify pre-sleep autonomic modulation. Measurements in which patients have fallen asleep are excluded from the analysis.
2. A nocturnal 6-h segment beginning after sleep onset. We choose this definition of the nocturnal HRV assessment for multiple reasons. According to guidelines, durations of recordings have to be of equal length [135]. Nocturnal measurement segments of 4 or 6 h are frequently used in studies [136, 137]. In such trials, the starting point of the measurement period is usually defined by either sleep onset (identified by EEG or sleep diary) or a fixed time (e.g., 00:00). Since sleep onset is associated with increased parasympathetic modulation [138], we use sleep onset defined by polysomnography to mark the starting point of the segment to be analyzed. Using this starting point avoids potential bias which might be introduced by inter-individual differences in sleep onset latency and chronotype when using a fixed time. We chose a 6-h segment as it offers a more extended measurement period than 4 h. Moreover, 6 h is commonly considered the cut-off for objective short sleep duration [139, 140] and thus can be understood as a minimum sleep requirement for most individuals. Nocturnal HRV is dynamic, as non-REM sleep (primarily during the first half of the night) and REM sleep (primarily during the second half of the night) are characterized by parasympathetic and sympathetic predominance, respectively [138, 141]. We split the 6-h period into hourly segments, which allows the dynamic mentioned above to be partially captured.
3. A short-term post-sleep segment of 5 min beginning after the last awakening and before standing up to quantify post-sleep autonomic modulation. Patients are instructed to lie supine in bed after awakening for 5 min (using a timer). The post-sleep measurement has the advantage of limiting external factors which influence HRV.

The time- and frequency-domain measures which we calculate are described in Table 3. Low (LF) and high frequency (HF) power in normalized units (LFnu and HFnu) will not be reported. The rationale for this deviation from guidelines is that LFnu, HFnu, and LF/HF ratio have been shown to carry algebraically and therefore physiologically redundant information [142, 144]. Reporting of data collection, analysis, cleaning, and calculation will follow the GRAPH guidelines [145]. The percentage of beats identified as artifacts will be reported for both groups for each segment (median and inter-quartile range). Measurements with > 5% artifacts will be excluded from analysis.

**Table 3:** HRV parameters

Method	Measure of variability	Calculation of variable	Physiological mechanism
**Time-domain methods**			
Statistical	RMSSD	Root mean square of successive differences of NN intervals	Short-term components of HRV, vagal modulation
	SDNN	Standard deviation of the of all NN intervals	Overall HRV, cyclic components responsible for HRV
	SDANN	Standard deviation of the averages of NN intervals in all 5-min segments of the entire recording (only for nocturnal HRV)	Long-term components of HRV
**Frequency domain methods**			
Lomb-Scargle Periodogram and Fast Fourier transformation	TP	Total power: power density spectrum in the frequency range of 0.00001 to 0.4 Hz [ms^2^]	Overall HRV
	LF	Low-frequency power: power density spectrum in the frequency range of 0.04 to 0.15 Hz [ms^2^]	Sympathetic and vagal activity, baroreflex activity (vasomotor tone)
	HF	High-frequency power: power density spectrum in the frequency range of 0.15 to 0.40 Hz [ms^2^]	Vagal modulation
	LF/HF	Ratio LF [ms^2^]/HF [ms^2^]	Sympathetic and vagal modulation

Calculations are based on [135, 142]. Descriptions of physiological mechanisms are based on [142, 143]

An ongoing debate in the literature concerns the choice of method for spectral analysis. Fast Fourier transform (FFT) using Welch’s periodogram and autoregressive modeling (AR) seem to be the most frequently used methods for power spectral analysis [135, 143]. Another method to estimate power spectral density is the Lomb-Scargle periodogram (LSP) [146, 147]. LSP has numerous advantages when compared to FFT and AR. LSP is designed to estimate the power density spectrum directly from the unevenly sampled tachogram. AR and FFT, on the other hand, need to be interpolated and resampled to fulfill the prerequisite of evenly sampled data. Resampling leads to over- and under-estimation of LF and HF, respectively [148, 149]. FFT and AR require a trade-off between frequency resolution and time resolution (statistical stability) when choosing window length [150] and model order [151], respectively. In contrast, LSP makes no assumptions of models. Investigations have shown that LSP is more accurate [152–154], is less noisy [154], has higher reliability [155], and is more sensitive to physiological changes [155–157] when compared to FFT. Consequently, multiple authors have suggested LSP as the method of choice for spectrum analysis of HRV [152, 158]. For these reasons, we estimate power spectral density using LSP smoothed with a moving average filter (width 0.02 Hz) in this trial. To enable comparison with other studies, we also calculate Welch’s FFT and will report it as a sensitivity analysis.

We perform ECG pre-processing and HRV analysis using Kubios HRV (University of Eastern Finland, Kuopio, Finland) [159]. This software has been shown to have perfect intra-class correlation coefficients (ICC = 1.000) across HRV variables measured during different postures when compared to two other software [120]. QRS detection is based on the Pan-Tompkins algorithm [160], including bandpass filtering. Beat detection is visually inspected. Erroneously detected RR fiducial points are corrected by manual editing of R-wave. Artifacts are identified using the automatic artifact correction algorithm [161]. This algorithm has been shown to detect ectopic beats with an accuracy of 97%. Ectopic beats are replaced by phantom beats using cubic spline interpolated RR values. Correction of aberrant RR intervals using cubic spline interpolation has been shown to perform as well as other correction methods for frequency analysis [162]. Detrending, i.e., removal of slow-trend and non-linear trend components is performed using the smoothness priors approach [163] with λ = 500 and f_c_ = 0.035 Hz (thus not affecting the lower band of LF). Frequency-domain variables estimated by LSP are based on de-trended RR series. Frequency-domain variables estimated by FFT with Welch’s periodogram are based on de-trended as well as interpolated (i.e., resampled) RR series. We employ the following parameters for power spectral density estimation using FFT. RR series are resampled to obtain an evenly sampled time series using a cubic spline interpolation with a rate of 4 Hz. A Hann window with a width of 60 s and 50% overlap is used (corresponding to 240 samples). These window parameters are chosen to balance the requirement of stationarity and frequency resolution resulting in an average of 9 and 119 FFT spectra for 5 min and hourly segments, respectively, with a frequency resolution of 0.025 Hz. Singh et al. [150] have shown that these approximate parameters produce a good spectral estimate (smooth with clear peaks).

Pulse transit time is calculated using ECG and pulse waveform from photoplethysmography. Blood pressure (BP) is calculated using the pulse transit time. This method has been validated [164] according to the European Society of Hypertension International Protocol (ESH-IP) revision 2010 criteria [165]. Accordingly, we perform a single initial calibration measurement (manual cuff-based method, contralateral arm of photoplethysmography, sitting position). Two significant advantages of this method are the continuous measurement of BP and elimination of cuff inflations. The latter is poorly tolerated by patients, causes awakenings, and may affect the validity of BP measurements [166]. BP levels differ between non-REM and REM sleep [167]. Therefore, mean systolic, mean diastolic, and mean arterial pressures will be reported separately for total sleep time, non-REM sleep, and REM sleep.

#### Subjective sleep-related measurements

Subjective pre-sleep arousal has been shown to be increased in primary insomnia [168] and seems to partially mediate the relationship between depressive symptoms and daytime fatigue [169]. We use the German version of the Pre-Sleep Arousal Scale to assess cognitive and somatic pre-sleep arousal. Eight and seven items load onto the factors somatic (Cronbach’s α = 0.80) and cognitive (Cronbach’s α = 0.94) arousal, respectively [170]. Items are scored on a five-point Likert scale (*not at all* to *extremely*) and summed up for each factor separately.

We measure subjective sleep quality of the baseline and post-intervention night using the revised *Schlaffragebogen A*, as recommended by guidelines [171]. Twenty-five items load onto five factors: sleep quality, recuperation after sleep, calmness before sleep, exhaustion before sleep, and psychosomatic symptoms during sleep. Internal consistency, factor structure, and validity have been demonstrated in numerous populations [172].

The clinical relevance of excessive daytime sleepiness is highlighted above (cf. rationale for ESS questionnaire). State sleepiness is recorded four times (0800, 1200, 1600, 2000 h) on the day after the experimental condition to gauge the effects on daytime somnolence. The Stanford Sleepiness Scale is a single item questionnaire assessing the degree of momentary sleepiness on a seven-point scale [173]. Adequate psychometric properties have been demonstrated [174].

#### Expectancy and credibility

Insomnia treatment guidelines stress the importance of contextual factors such as patient preference and satisfaction when choosing the most suitable therapy [88, 175]. Credibility can increase expectancy [176]. The latter has been shown to influence outcomes in depression and other disorders [177]. Since patients cannot be blinded in exercise studies, it is especially important to consider expectancy. We assess credibility and expectancy on day three (i.e., before randomization) using two items (adapted from [178, 179]): “At this point, how logical does the therapy offered to you seem?”, “At this point, how successfully do you think this treatment will be in reducing your insomnia symptoms?”. Patients rate these items on a four-point Likert scale (*not at all* to *very*).

#### Randomization and blinding

A non-deterministic minimization algorithm is used to assign interventions. Allocation to intervention or control group is done using the open source software for online minimization (Oxford Minimization and Randomization, OxMaR) [180]. All necessary data for minimization is collected using surveys integrated into the eCRF. The required data are entered in the web-based randomization software by the study nurses. Upon confirmation that the data are correct, the study participant is allocated to a group, the allocation is saved in a central database, and an e-mail containing the allocation is sent to the PI, the study coordinator, and to the person submitting the participant. Allocation concealment consists of four aspects: (1) requesting randomization after baseline measurement, (2) use of a random element, (3) requesting allocation for participants by two different study nurses, and (4) not disclosing full details of minimization to study nurses in accordance with the SPIRIT guideline [67]. We will publish a detailed description of the minimization scheme with the results of the trial.

It is impossible to blind participants in exercise trials. However, we prevent detection bias though objective sleep measures which are assessed by two blinded and independent assessors. Blinding is ensured by replacing the subject ID with a second unique ID number. The list matching these IDs is not accessible to the raters.

#### Intervention and control condition

##### Aerobic exercise (intervention)

Patients allocated to the intervention group perform a single bout of supervised aerobic exercise. The starting time is approximately 1645 h. The exercise mode is a bicycle ergometer (ergoselect 200, Ergoline, Bitz, Germany). After a warm-up period of 5 min, during which the intensity is gradually increased, patients maintain an intensity of 80% of the individual anaerobic threshold for 30 min. The intensity level is chosen based on clinical experience that this corresponds to an approximate rate of perceived exertion of 13 (on a scale from 6 to 20) in this population. The duration of exercise corresponds to physical activity recommendations [181].

##### Control condition

At the same time as individuals performing the exercise intervention, individuals allocated to the control group are placed in a room which is comparable to that of the exercise group concerning light, temperature, and absence of music. The control group is asked to remain seated and read magazines.

The rules and schedules of the inpatient rehabilitation clinic (e.g., timing of meals, consumption of multimedia, and alcohol) limit the variability of many behavioral aspects which could influence sleep. Occasional smokers are asked to refrain from smoking after dinner on the days of polysomnographic assessment. Chronic smokers are not asked to abstain from smoking as this might be an additional stressor.

##### Adherence and other outcomes of interest

We assess the implementation of the intervention with continuous measurement of Watts and heart rate using a Polar® H7 chest strap (Polar OY, Finland). We measure heart rate throughout the intervention period, including 3 min post-exercise. We measure perceived exertion using the Borg scale (6–20) [93]. All subjects complete a questionnaire on their current mood immediately before and at the end of the control condition as well as the exercise intervention. The *Befindlichkeitsskala* has adequate psychometric properties [182] and is considerably more economical than other comparable measures [183]. The questionnaire consists of 40 items on a five-point Likert scale (*not at all* to *very much*). Items load onto eight subscales (with five items each): activity (Cronbach’s α = 0.82), elation (Cronbach’s α = 0.81), contemplation (Cronbach’s α = 0.70), calmness (Cronbach’s α = 0.78), fatigue (Cronbach’s α = 0.88), depression (Cronbach’s α = 0.80), anger (Cronbach’s α = 0.86), and excitement (Cronbach’s α = 0.73) [182]. Contamination through any or additional physical activity (depending on the allocation) is assessed using a wrist-worn accelerometer (on non-dominant hand) on the days prior to and after the sleep assessments. The wrist-worn accelerometer vivofit®2 (Garmin, Schaffhausen, Switzerland) validly assesses steps in various walking conditions [184]. Although adequate blinding cannot be achieved by design in exercise studies, this allows for a partial assessment of performance bias.

##### Concomitant, ancillary, and post-trial care

The trial takes place in the first 5 days of the patient’s psychosomatic in-patient rehabilitation. The rehabilitation programme entails different therapies, including exercise therapy. Patients included in the study are asked to refrain from exercise except as defined by the protocol on the days of testing. Patients are explicitly made aware of this aspect before enrollment. Ancillary and post-trial care is provided throughout the in-patient rehabilitation program, i.e., on average for 4 weeks after completion of the study.

##### Adverse events

We assess adverse events through a questionnaire. We ask patients in both groups whether they experience adverse effects on a five-point Likert scale (*not at all* to *very*) using the following categories immediately after the intervention and the following morning:

- Pain (if yes, location)
- Dizziness
- Cardiovascular symptoms (e.g., angina symptoms, cyanosis, pallor)
- Respiratory symptoms (e.g., wheezing)
- Nausea
- Falls (yes or no)
- Other (to be described)

Unplanned termination of participants and the reasons thereof (if participant proactively gives one) will also be reported.

### Data management

#### Data collection

We use castor electronic data capture software for data collection and data management [185]. This software complies with Good Clinical Practice guidelines and the European Data Protection Directive. A unique numeric subject ID is assigned to each participant to conceal the identity of participants in the database. The file which links the numeric subject ID to the participant information is kept in a password-protected folder in an encrypted digital file and on paper in a locked cabinet. These digital and paper files are exclusively stored at the study site. Only the study nurses, the study coordinator, and the principal investigator have access to this information. Regulatory agencies (i.e., ethics committee) will also be granted access upon request. Patient data are collected using electronic case report forms (eCRF). Range and dependency checks are implemented into the eCRF and completeness checks of data for each participant are performed during the trial. The eCRF can be found in Additional file 3. All study personnel have been trained in measurement procedures and data collection according to the case report form using standard operating procedures to ensure standardized data collection. Data from the polysomnography are uploaded to the CASTOR platform using the subject ID to match data.

#### Security, storage, and access

Study nurses, the study coordinator, the principal investigator, and monitors can access the password-protected database. The principal investigator and study coordinator define user accounts and user rights according to their responsibilities, e.g., authorization for data changes. All changes in the eCRF are saved in data trails, audit trials, and edit trails, (including reasons for changes). The data are stored for 15 years. The study coordinator and principal investigator will have full access to the trial data.

#### Data monitoring and audits

Due to the risk stratification of this trial, the need for a data-monitoring committee is waived. Central data monitoring is performed using the built-in modules of the CASTOR software. No interim analyses are planned. Monitoring of regulatory files, study processes, and data is conducted in four visits: before enrollment of the first patient, after enrollment of the first patient, after enrollment of 50% of patients, and after last patient last visit. Monitoring is done by the Clinical Trial Unit, Basel, Switzerland (i.e., an organization independent from the investigator and sponsor).

### Statistical methods

#### Sample size calculation

The theoretical rationale for the analysis of the primary outcome, using an ANCOVA model, is outlined above (see the “Polysomnography (baseline and follow-up)” section). Furthermore, minimization necessitates adjustment for minimization factors [186]. Sample size calculation was performed according to the procedure defined by Borm et al. [187]. The allocation ratio is 1:1. The expected treatment effect is based on the work of Passos et al. [63], who found a standardized mean difference in sleep efficiency of 0.53. The estimate is based on this publication because it is the only one known to the authors which (1) evaluated the *acute* effect of (2) a similar intervention (i.e., moderate aerobic exercise) (3) in individuals with sleep disorders. Due to the well-documented ceiling and floor effects [188], meta-analyses concerning predominantly healthy individuals (explicitly excluding individuals with mental disorders) [54] are of no use. Meta-analyses including individuals with sleep complaints are limited to the analysis of the *chronic* effects of exercise on sleep [55, 189]. With a power of 0.8 and a two-sided alpha of 0.05, 57 subjects would be required for each group using a *t*-test. According to the method of Borm et al., this sample size can be multiplied by a ‘design factor’ of (1 − ρ^2^), where ρ is the correlation coefficient between baseline and follow-up outcome [187]. Despite contacting other researchers, the authors are not aware of any previous study which have analyzed this aspect. Hence, we need to make an estimate. Recommended values for imputation of ρ vary between 0.5 (if variances are equal at pre- and post-measurements, ρ is at least 0.5) and 0.7 [190, 191]. We use a conservative estimate and let ρ = 0.5. This leads to a design factor of 0.75 (1 − 0.5^2^ = 0.75). Hence, the sample size needed per group is 43 (57 × 0.75 = 42.75). Due to the short-term nature of this study, we expect approximately half the dropout rate of trials investigating the chronic effects of exercise in patients with depression [192]. Thus, we anticipate 7% dropouts. Therefore, the total sample size is 92 (2 × 43 × 1.07 = 92).

#### Analysis of the primary outcome

The main aim of this trial is to analyze the acute effect of aerobic exercise on the subsequent night’s sleep efficiency measured by polysomnography. To this end, we will compute a one-way ANCOVA with baseline sleep efficiency and minimization factors as covariates, intervention as the independent variable, and post-exercise sleep efficiency as the dependent variable. Clinical significance will be determined using the criteria defined in the American Academy of Sleep Medicine Clinical Practice guideline for the Pharmacological Treatment of Chronic Insomnia in adults. Thereby, an absolute change in sleep efficiency of ≥ 5% is deemed clinically relevant. We define responders as individuals who have an increase in sleep efficiency of ≥ 5% from baseline to post-intervention. We will report the number, the proportion, and the odds ratio of responders as well as the number needed to treat. In order to reduce attrition bias to a minimum, all analyses will follow the intention-to-treat framework.

Sensitivity analyses for the primary outcome will be performed to gauge the influence of several factors: outliers (defined as < Quartile 1 − 1.5 × Interquartile range; > Quartile 3 + 1.5 × Interquartile range), per-protocol analysis (to reflect optimal adherence to treatment), missing data (analysis of complete data only), excluding minimization factors (age, sex, PHQ-9 score, and Pittsburgh Sleep Quality Index score), chronotypes, smoking status, and use of beta-blockers. We will report all results of sensitivity analyses. We will replace missing data using multiple imputation with the *mi* package in R [193]. The quantity of missing data will be reported.

#### Analysis of secondary outcomes

Secondary outcomes will also be analyzed using ANCOVA models when variables have been assessed at baseline and follow-up. Hourly segments of nocturnal HRV will be analyzed using a linear mixed model with subject as random effect, adjusting for baseline and minimization factors. Acute daytime sleepiness will be assessed using repeated measures ANOVA with Benjamini-Hochberg [194] corrected post hoc paired sample *t*-tests. Measurements where only post-intervention group-comparisons are possible will be analyzed using *t*-tests. The threshold for statistical significance is set at *p* < 0.05. The authors point out that the secondary analyses are not adjusted for multiple testing and are of exploratory nature.

### Ethics and dissemination

#### Ethics approval

The Ethics Committee East Switzerland, St. Gallen, Switzerland, has approved the study (EKOS 18/089). We registered the trial with the ClinicalTrials.gov database (NCT03673397) on September 17, 2018. No protocol amendments have been made. Approval for any potential future amendments will be obtained from the local ethics committee.

#### Dissemination policy

We will disseminate trial results to all stakeholders, e.g., participants (if they choose to receive these at enrollment), physicians, and study nurses. Results will be published in a peer-reviewed journal and presented at conferences as well as invited talks. Authorship on peer-reviewed publications will be based on contributions toward design, fundraising, data collection, analysis, and manuscript preparation. De-identified (i.e., coded) individual participant data that underlie the results of published articles, including data dictionaries, will be available upon request under the creative commons license CC-BY. Requests will only be granted for use in individual participant data meta-analysis which has been approved by an independent review committee. Exceptions to these rules are reserved within the context of peer-reviewed publications, provided that data integrity remains intact. Data will be provided upon request immediately after publication of peer-reviewed articles with no end date. Requests should be sent to the e-mail address detailed in the following Dataverse repository (10.7910/DVN/WASN36).

## Discussion

The goal of this two-arm parallel group, individually randomized, single-blind, controlled trial is to assess the superiority of an acute bout of aerobic exercise compared to no intervention in improving the subsequent night’s sleep efficiency in patients with a primary diagnosis of depression. To the knowledge of the authors, this is the first trial to investigate the acute effects of exercise on sleep in this population.

The main strength of this study is the objective measurement of sleep. Moreover, multiple secondary outcomes were carefully selected to provide clinicians, patients, and policy makers with a comprehensive picture of the effects this intervention may have. Explicit inclusion of patients with comorbidities should enhance the external validity of this study. Accordingly, we provide extensive baseline characterization.

The main limitation of this trial is the restricted polysomnographic EEG montage. More detailed analyses, such as spectral EEG analysis, will therefore not be possible. Extensive discussions in our research group resulted in an explicit trade-off between the patients’ discomfort (number of EEG channels) and feasibility of recruitment. The restricted EEG montage will help to recruit the necessary number of patients in an adequate period in this clinical setting. The pre-selection of patients who are treated in this rehabilitation clinic might pose a further limitation. Extensive baseline characterization will help readers identify limits to external validity. Patients cannot be blinded against allocation in exercise trials. We try to overcome this limitation through various measures (see above).

There is compelling evidence for the effectiveness of various therapies for both insomnia and depression. Nevertheless, many of these therapies have shortcomings. Development of further therapeutic options, which can be administered in addition to these therapies, are therefore needed. Current literature suggests that acute aerobic exercise might improve both depression and insomnia. Although chronic aerobic exercise is included in sleep hygiene recommendations, there is uncertainty concerning the acute effects of aerobic exercise on sleep in patients with depression. This trial aims to close this gap in the literature as well as to help the increasing number of patients with depression.

## Trial status

We registered this trial on September 17, 2018, in the ClinicalTrials.gov database (NCT03673397). All items of the World Health Organization Trial Registration Data Set can be found in the ClinicalTrials.gov registration. This is the first version of the protocol, i.e., we have made no amendments. Recruitment began on September 24, 2018. Recruitment is expected to be completed by October 2019.

## Additional files

Additional file 1: SPIRIT checklist. (PDF 171 kb)

Additional file 2: Original consent form in German. (PDF 561 kb)

Additional file 3: Electronic case report form (eCRF). (PDF 504 kb)

## Data Availability

The dataset supporting the conclusions of this article is available in the Dataverse repository (10.7910/DVN/WASN36). De-identified (i.e., coded) individual participant data that underlie the results of published articles, including data dictionaries, will be available upon request under the creative commons license CC-BY. Requests will only be granted for use in individual participant data meta-analysis which has been approved by an independent review committee. Exceptions to these rules are reserved within the context of peer-reviewed publications, provided that data integrity remains intact. Data will be provided upon request immediately after publication of peer-reviewed articles with no end date. Requests should be sent to the e-mail address detailed in the Dataverse repository mentioned above.

## Abbreviations

ANCOVA: Analysis of covariance
AR: Autoregressive modeling
BMI: Body mass index
BP: Blood pressure
CBT-I: Cognitive behavioral therapy for insomnia
ECG: Electrocardiogram
eCRF: Electronic case report form
EEG: Electroencephalogram
EOG: Electrooculogram
FFT: Fast Fourier transform
HF: High frequency power
HRV: Heart rate variability
ICD-10: International Statistical Classification of Diseases and Related Health Problems, 10th revision
LF: Low frequency power
LSP: Lomb-Scargle periodogram
N1–N3: Stage 1–3 sleep
NA: Number of awakenings
ODI: Oxygen desaturation index
PHQ: Patient Health Questionnaire
REM: Rapid eye movement
SE: Sleep efficiency
SOL: Sleep onset latency
SPIRIT: Standard Protocol Items: Recommendations for Interventional Trials (SPIRIT)
TRT: Total recording time
TST: Total sleep time
WASO: Wake after sleep onset
