# Supplementary material for: The acute effects of aerobic exercise on sleep in patients with depression: study protocol for a randomized controlled trial
Source: Trials. 2019 Jun 13;20:352. doi: 10.1186/s13063-019-3415-3 (PMC6567535; doi:10.1186/s13063-019-3415-3)
Supplement: Supplementary file 2 — Original consent form in German. (PDF 561 kb) [file 13063_2019_3415_MOESM2_ESM.pdf]

OBERWAID
DAS HOTEL. DIE KLINIK.

Effekte eines Ausdauertrainings auf den Schlaf bei Patienten mit Depression – EASED Studie

(Originaltitel: The acute effect of aerobic exercise on sleep in patients with depression – EASED trial)

Diese Studie ist organisiert durch: OBERWAID AG

Sehr geehrte Dame, sehr geehrter Herr,

Wir möchten Sie anfragen, ob Sie an einer Klinischen Studie teilnehmen wollen. Im Folgenden wird Ihnen dieses Studienvorhaben dargestellt: zunächst in einer kurzen Zusammenfassung, damit Sie wissen, um was es geht, anschliessend in einer detaillierten Beschreibung.

Zusammenfassung

1	**Ziel der Studie**<br>Wir möchten Sie hiermit bitten, an unserer Klinischen Studie teilzunehmen. Die Studie untersucht Schlafstörungen bei Patienten mit Depression. Wir machen diese Studie, um die Wirkung eines Ausdauertrainings auf den Schlaf zu untersuchen.
2	**Auswahl**<br>Sie leiden unter einer Depression. Deshalb lassen wir Ihnen diese Informationsschrift zukommen. Nicht teilnehmen können Patienten, welche regelmässig Schlafanstossende Medikamente nehmen.
3	**Allgemeine Informationen zur Studie**<br>Aktuelle Forschung weist darauf hin, dass Schlafstörungen bei Depression sehr relevant sind. Daher kommt der Behandlung von Schlafstörungen ein besonderer Stellenwert zu. Ausdauertraining scheint bei Gesunden und bei Patienten mit einer isolierten Schlafstörung (d.h. keine zusätzliche Depression), den Schlaf positiv zu beeinflussen. Bislang gibt es aber nur wenige wissenschaftliche Erkenntnisse zur Wirkung von Training auf den Schlaf bei Patienten mit Depression. Daher untersuchen wir wie sich ein einzelnes aerobes Ausdauertraining auf den Schlaf in der darauffolgenden Nacht bei Patienten mit Depression auswirkt.
4	**Ablauf**<br>Patienten werden per Zufall in eine von zwei Gruppen eingeteilt. Eine Gruppe wird ein 30-minütiges moderates Ausdauertraining auf dem Fahrradergometer machen. Die andere Gruppe wird Zeitschriften lesen. In dieser Studie werden nur etablierte klinische Verfahren und Messmethoden eingesetzt. Insgesamt werden 92 Patienten im Verlauf von ca. einem Jahr an der Studie teilnehmen.<br>Für die Studie werden in den ersten 5 Tagen Ihres Aufenthaltes in der Oberwaid zusätzlichen Messungen und ein spezifisches Ausdauertraining gemacht. Diese zusätzlichen Messungen umfassen Fragebögen, eine Aktivitätsmessung und eine weitere Messung des Schlafes (Polysomnografie). Die Studie ist am Montagabend (5 Tage) nach Eintritt abgeschlossen.
5	**Nutzen**<br>Sie haben durch die Teilnahme an der Studie einen sehr geringen Nutzen. Sie können aber helfen die Behandlungsqualität für Patienten mit dieser Erkrankung zu verbessern. Wir werden Sie über Ihre individuellen Resultate und über das Gesamtergebnis der Studie

	informieren, falls sie dies wünschen. Eine zusätzliche materielle Entschädigung ist nicht vorgesehen.
6	**Rechte**<br>Sie entscheiden freiwillig, ob Sie an der Studie teilnehmen wollen oder nicht. Ihre Entscheidung hat keinen Einfluss auf Ihre medizinische Behandlung und Sie müssen diese Entscheidung nicht begründen.
7	**Pflichten**<br>Wenn Sie teilnehmen, bitten wir Sie, bestimmte Anforderungen einzuhalten (z.B. zusätzliches Training am ersten Wochenende in der Klinik zu vermeiden).
8	**Risiken**<br>Das vorgesehene Ausdauertraining birgt kaum Risiken. Risiken des Trainings für Ihr Herzkreislaufsystem werden vorgängig durch einen Arzt abgeklärt. Sie werden während des Trainings sicherlich schwitzen und sich danach etwas verausgabt fühlen. Ansonsten bestehen durch das Training keine Risiken.
9	**Andere Behandlungsmöglichkeiten**<br>Ihr Arzt wird Sie beraten, welche anderen Möglichkeiten zu Ihrer Behandlung bestehen.
10	**Ergebnisse**<br>Bei Studienergebnissen während der Studie werden Sie informiert, wenn diese Ergebnisse für Sie gesundheitlich wichtig sind. Falls Sie dies nicht möchten, informieren Sie bitte Ihre Prüfärztin.
11	**Vertraulichkeit von Daten**<br>Wir halten alle gesetzlichen Regeln des Datenschutzes ein und alle Beteiligten unterliegen der Schweigepflicht. Ihre persönlichen und medizinischen Daten werden verschlüsselt verwendet und geschützt. Die Daten werden für andere Forschungsprojekte weiter verwendet, wenn Sie Ihr separates Einverständnis dafür geben.
12	**Rücktritt**<br>Sie können jederzeit von der Studie zurück treten und nicht mehr teilnehmen. Die bis dahin erhobenen Daten werden noch ausgewertet.
13	**Entschädigung**<br>Sie erhalten keine Entschädigung.
14	**Haftung**<br>Die Haftplicht der OBERWAID AG kommt für allfällige Schäden im Rahmen der Studie auf.
15	**Finanzierung**<br>Die Studie wird von der OBERWAID AG bezahlt.
16	**Kontaktperson:**<br>Sie erhalten jederzeit auf alle Ihre Fragen Auskunft.<br>MSc Gavin Brupbacher<br>Studienkoordinator & Prüfperson<br>Rorschacher Strasse 311<br>9016 St.Gallen<br>gavin.brupbacher@oberwaid.ch<br>071 282 07 64<br><br>Dr. med. Hildburg Porschke<br>Sponsor-Investigator & Prüfärztin<br>Rorschacher Strasse 311<br>9016 St.Gallen<br>hildburg.porschke@oberwaid.ch<br>071 282 07 13

Detailliertere Information

1. Ziel der Studie

Wir wollen mit dieser Studie untersuchen, ob ein einmaliges Ausdauertraining sich positiv auf den Schlaf auswirkt.

2. Auswahl

Es können alle Personen teilnehmen, die an einer Depression leiden und sich in stationärer psychosomatischer Behandlung in der Oberwaid befinden. Ausserdem müssen sie 18 bis 65 Jahre alt sein. Nicht teilnehmen hingegen dürfen Personen, die u.a. regelmässig Schlafmedikamente nehmen oder kein Ausdauertraining machen können.

3. Allgemeine Informationen

- Aktuelle Forschung weisst darauf hin, dass Schlafstörungen bei Depression sehr relevant sind. Sie können den Krankheitsverlauf negativ beeinflussen. Daher kommt der Behandlung von Schlafstörungen besonderen Stellenwert zu.
- Ausdauertraining scheint bei Gesunden und bei Patienten mit einer isolierten Schlafstörung (d.h. keine zusätzliche Depression), den Schlaf positiv zu beeinflussen. Bislang gibt es aber nur wenige wissenschaftliche Erkenntnisse zur Wirkung von Training auf den Schlaf bei Patienten mit Depression. Daher untersuchen wir wie sich ein einzelnes aerobes Ausdauertraining auf den Schlaf in der darauffolgenden Nacht bei Patienten mit Depression auswirkt.
- Dies ist eine randomisierte kontrollierte einfachblinde Studie (siehe auch Glossar, Punkt 17). Randomisiert bedeutet, dass die Zuteilung der Patienten in eine der beiden Behandlungsgruppen (Ausdauertraining oder Kontrollgruppe) nach dem Zufallsprinzip erfolgt. Ihre Chance in eine der beiden Gruppen eingeteilt zu werden liegt bei 50%. Kontrolliert heisst, dass die Resultate der Ausdauertrainings-Gruppe mit den Resultaten der Kontrollgruppe verglichen werden. Einfachblind meint hier, dass die Forscher, welche die Schlafmessung analysieren, die Gruppenzuteilung nicht kennen.
- Wir machen diese Studie so, wie es die Gesetze in der Schweiz vorschreiben. Ausserdem beachten wir alle international anerkannten Richtlinien. Die zuständige Kantonale Ethikkommission hat die Studie geprüft und bewilligt.
- Eine Beschreibung dieser Studie finden Sie auch auf der Internetseite des Bundesamtes für Gesundheit: www.kofam.ch

4. Ablauf

- Die Studie findet schwerpunktmäßig am ersten Wochenende Ihres Aufenthaltes statt. Damit möchten wir sicherstellen, dass Sie genügend Zeit zum Ankommen in der Klinik haben. Einige Untersuchungen und Messungen finden bereits in den ersten drei Tagen (Mittwoch - Freitag) im Rahmen des normalen stationären Aufenthaltes statt. Die Studie ist am ersten Montagabend (5 Tage) nach Eintritt abgeschlossen.
- Die Abbildung 1 (siehe nächste Seite) zeigt den Ablauf der Studie. Einige Messungen sind Teil der normalen stationären psychosomatischen Rehabilitation: der submaximale Ausdauertest, die erste Schlafmessung sowie einige Fragebögen. Gewisse Ein- und Ausschlusskriterien werden im Rahmen der Eintrittsuntersuchungen, welche auch Teil der normalen Behandlung sind, erfasst. Studienspezifische Messungen werden erst nach dem Unterschreiben der Einverständniserklärung durchgeführt.

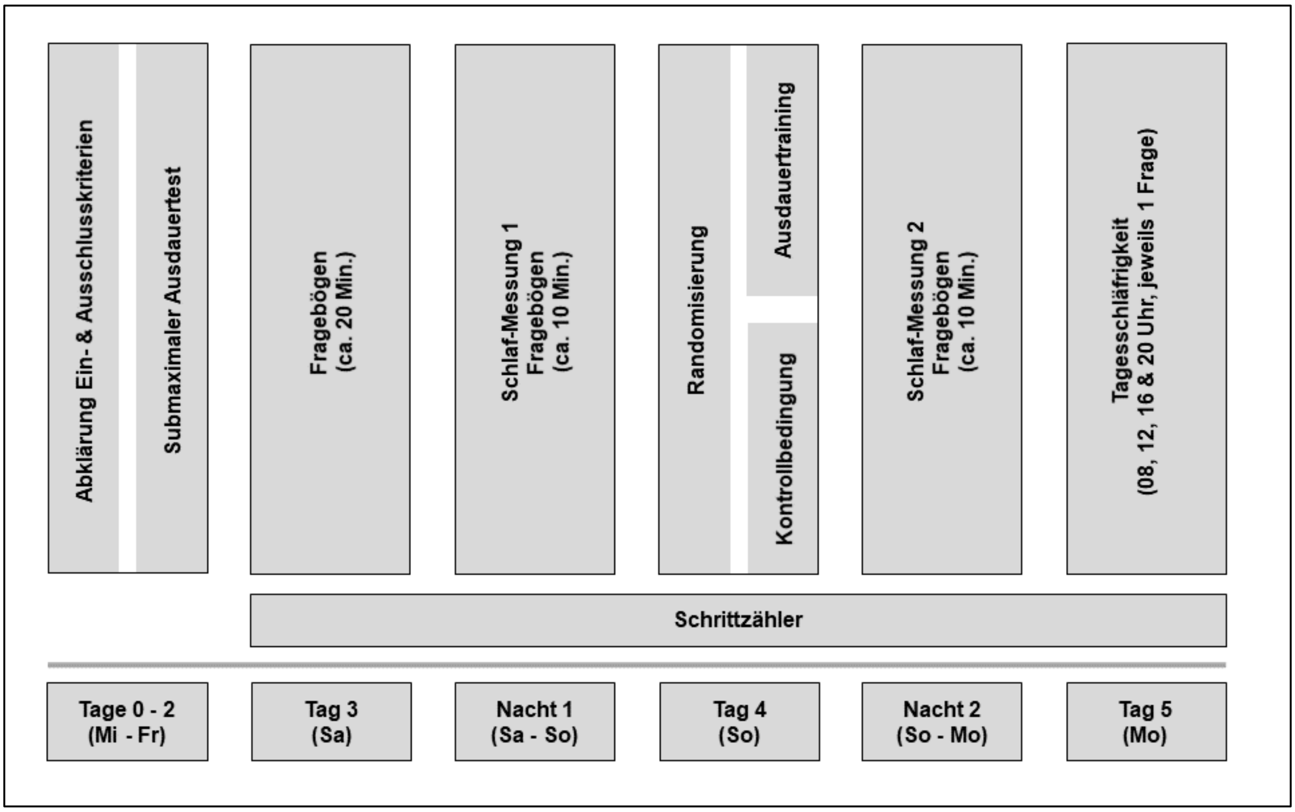

Abbildung 1: Ablauf der Studie

- Wenn Sie an der Studie teilnehmen können und wollen, wird am Freitag ein submaximaler Ausdauertest gemacht. Submaximal bedeutet, dass Sie nicht an Ihre Leistungsgrenze gehen.
- Am Samstag füllen Sie einige Fragebögen aus. Dies dauert ca. 20 Minuten.
- Von Samstag auf Sonntag findet die erste Messung ihres Schlafes statt. Mittels der sog. Polysomnografie werden Herzrhythmus (EKG), Sauerstoffsättigung (Infrarot-clip am Finger), Muskelaktivität (EMG) und Hirnströme (EEG) gemessen. Am Sonntagmorgen werden Fragebögen beantwortet (ca. 10 Minuten). Diese Messungen geben uns Aufschluss darüber wie gut sie geschlafen haben.
- Bitte beachten Sie: Für die Messung der Sauerstoffsättigung während der Schlafmessung soll am Zeigefinger kein Nagellack aufgetragen oder künstlicher Fingernagel angebracht sein.
- Am Sonntagnachmittag werden Sie nach dem Zufallsprinzip einer der beiden Gruppen zugeteilt (Randomisierung). Die Ausdauertrainingsgruppe wird einmalig 30 Minuten auf einem Fahrradergometer bei einer individuell abgestimmten mittleren Intensität trainieren. Ein solches Training entspricht den nationalen und internationalen Leitlinien zur Behandlung von Depression und zur Gesunderhaltung des Körpers. Die Kontrollgruppe wird 30 Minuten Zeitschriften lesen.
- Von Sonntag auf Montag findet die zweite Messung ihres Schlafes statt, inkl. Fragebögen (ca. 10 Minuten) am Montagmorgen.
- Am Montag werden Sie um 08:00, 12:00, 16:00 und 20:00 Uhr zur ihrer Tagesschläfrigkeit befragt (jeweils eine Frage).
- Die gesamte Studie findet ausschliesslich in der OBERWAID statt.
- Die voraussichtliche Dauer der gesamten Studie ist ein Jahr. Gesamthaft werden 92 Patienten in die Studie eingeschlossen.

Es kann sein, dass wir Sie von der Studie vorzeitig ausschliessen müssen. Das kann geschehen, wenn medizinische Gründe gegen eine weitere Teilnahme an der Studie sprechen. In diesem Fall werden Sie zu ihrer Sicherheit abschliessend noch einmal untersucht.

5. Nutzen

Das Ausdauertraining könnte dazu führen, dass Sie in der darauffolgenden Nacht besser schlafen. Sie werden sonst persönlich keinen Nutzen von der Teilnahme an der Studie haben. Die Resultate können wichtig sein für andere, die dieselbe Krankheit haben.

6. Rechte

Sie nehmen freiwillig teil. Wenn Sie nicht mitmachen oder später Ihre Teilnahme zurückziehen wollen, müssen Sie dies nicht begründen. Ihre medizinische Behandlung/Betreuung ist unabhängig von Ihrem Entscheid gewährleistet. Sie dürfen jederzeit Fragen zur Studienteilnahme stellen. Wenden Sie sich dazu bitte an die Person, die am Ende dieser Information genannt ist.

7. Pflichten

Als Teilnehmer ist es notwendig, dass Sie

- sich an die notwendigen Vorgaben und Anforderungen der Studie durch den Prüfplan halten (z.B. kein zusätzliches Training am ersten Wochenende in der Klinik zu machen)
- Ihre Prüfärztin über den Verlauf der Erkrankung informieren und neue Symptome, neue Beschwerden und Änderungen im Befinden zu melden;
- Ihren behandelnden Arzt über die gleichzeitige Behandlung und Therapie bei einem anderen Arzt und über die Einnahme von Medikamenten, auch Medikamente der Komplementärmedizin informieren.

8. Risiken und Belastungen für die Teilnehmenden

- Die möglichen Risiken, welche durch eine Teilnahme an dieser Studie entstehen, sind minimal.
- Risiken für Ihr Herzkreislaufsystem werden vorgängig durch einen Arzt abgeklärt.
- Der Ausdauertest ist submaximal. Dies bedeutet, dass Sie nicht an Ihre Leistungsgrenze gehen. Im Test werden Ihnen lediglich einige Tropfen Blut am Ohrläppchen abgenommen.
- Das vorgesehene Ausdauertraining birgt kaum Risiken. Das Training findet unter der Aufsicht ausgebildeter Sportwissenschaftler statt. Eine körperliche Überforderung wird durch die individuelle Festlegung der Trainingsintensität praktisch ausgeschlossen. Zudem können Sie das Training jederzeit abbrechen.
- In sehr seltenen Fällen können die Klebeelektroden (zur Messung des Herzrhythmus, der Muskelaktivität und den Gehirnströmen) zu Hautirritationen führen.
- Es werden keine weiteren Risiken bei den anderen Untersuchungen erwartet.

9. Andere Behandlungsmöglichkeiten

Sie müssen bei dieser Studie nicht teilnehmen. Wenn Sie nicht mitmachen, hat dies keine Auswirkungen auf Ihre Behandlung. Schlafstörungen werden unabhängig von der Studienteilnahme mittels routinemässigen Abklärungen diagnostiziert und bei Bedarf entsprechend behandelt.

10. Ergebnisse aus der Studie

Die Prüfärztin wird Sie während der Studie über alle neuen Erkenntnisse informieren, die den Nutzen der Studie oder Ihre Sicherheit und somit Ihr Einwilligung zur Teilnahme an der Studie beeinflussen können. Sie werden die Information mündlich und schriftlich erhalten. Wenn Sie nicht informiert werden wollen, sprechen Sie bitte mit Ihrer Prüfärztin.

11. Vertraulichkeit der Daten

Für diese Studie werden Ihre persönlichen und medizinischen Daten erfasst. Nur sehr wenige Fachpersonen werden Ihre unverschlüsselten Daten sehen, und zwar ausschliesslich, um Aufgaben im Rahmen der Studie zu erfüllen. Bei der Datenerhebung zu Studienzwecken werden die Daten verschlüsselt. Verschlüsselung bedeutet, dass alle Bezugsdaten, die Sie identifizieren könnten (Name, Geburtsdatum), gelöscht und durch einen Schlüssel ersetzt werden. Die Schlüssel-Liste bleibt immer in der Institution. Diejenigen Personen, die den Schlüssel nicht kennen, können daher keine Rückschlüsse auf Ihre Person ziehen. Bei einer Publikation sind die

zusammengefassten Daten daher auch nicht auf Sie als Einzelperson rückverfolgbar. Ihr Name taucht niemals im Internet oder in einer Publikation auf. Manchmal gibt es die Vorgabe bei einer Zeitschrift zur Publikation, dass Einzel-Daten (sogenannte Roh-Daten) übermittelt werden müssen. Wenn Einzel-Daten übermittelt werden müssen, dann sind die Daten immer verschlüsselt und somit ebenfalls nicht zu Ihnen als Person rückverfolgbar. Alle Personen, die im Rahmen der Studie Einsicht in Ihre Daten haben, unterliegen der Schweigepflicht. Die Vorgaben des Datenschutzes werden eingehalten und Sie als teilnehmende Person haben jederzeit das Recht auf Einsicht in Ihre Daten.

Die Daten werden ausschliesslich verschlüsselt nach Holland versandt und dort für 15 Jahre aufbewahrt. Die Schlüssel-Liste bleibt in der Institution und Zugriff haben nur Hildburg Porschke (OBERWAID AG) und Gavin Brupbacher (OBERWAID AG). Der Sponsor ist dafür verantwortlich zu sorgen, dass im Ausland die gleichen Standards wie in der Schweiz eingehalten werden.

Es ist möglich, dass Ihre Daten für andere Untersuchungen zu einem späteren Zeitpunkt weiterverwendet werden oder später an eine andere Datenbank in der Schweiz oder ins Ausland für noch nicht näher definierte Weiterverwendung versandt und verwendet werden. Diese andere Datenbank muss die gleichen Standards einhalten wie die Datenbank zu dieser Studie. Für diese Weiterverwendung bitten wir Sie, ganz am Ende dieses Dokuments eine weitere Einwilligungserklärung zu unterzeichnen.

Möglicherweise wird diese Studie durch die zuständige Ethikkommission überprüft. Die Prüfärztin muss eventuell Ihre persönlichen und medizinischen Daten für solche Kontrollen offenlegen. Ebenso kann es sein, dass bei Schäden ausnahmsweise auch ein Vertreter der Versicherung Ihre Daten ansehen muss. Alle Personen müssen absolute Vertraulichkeit wahren.

12. Rücktritt
Sie können jederzeit aufhören und von der Studie zurücktreten, wenn Sie das wünschen. Die bis dahin erhobenen Daten werden noch verschlüsselt ausgewertet. Nach der Auswertung werden Ihre Daten anonymisiert, d.h. Ihre Schlüsselzuordnung wird vernichtet, so dass danach niemand mehr erfahren kann, dass die Daten ursprünglich von Ihnen stammten.

13. Entschädigung für Teilnehmende
Wenn Sie an dieser Studie teilnehmen, bekommen Sie dafür keine Entschädigung. Es entstehen Ihnen oder Ihrer Krankenkasse keine Kosten durch die Teilnahme.

14. Haftung
Die OBERWAID AG, die die Studie veranlasst hat und für die Durchführung verantwortlich ist, haftet für Schäden, welche Ihnen im Zusammenhang mit der getesteten Substanz oder Forschungshandlungen (z.B. Untersuchungen) entstehen könnten. Die Voraussetzungen und das Vorgehen dazu sind gesetzlich geregelt.
Falls Sie einen Schaden erlitten haben, so wenden Sie sich bitte an die Prüfärztin.

15. Finanzierung der Studie
Die Studie wird vollständig von der OBERWAID AG bezahlt.

16. Kontaktperson(en)
Bei Fragen, Unsicherheiten oder Notfällen, die während der Studie oder danach auftreten, können Sie sich jederzeit an eine dieser Kontaktpersonen wenden.

Gavin Brupbacher
Studienkoordinator & Prüfperson
Rorschacher Strasse 311
9016, St.Gallen
gavin.brupbacher@oberwaid.ch
071 282 07 64

Dr.med. Hildburg Porschke
Sponsor-Investigator & Prüfärztin
Rorschacher Strasse 311
9016, St.Gallen
hildburg.porschke@oberwaid.ch
071 282 07 13

Bei medizinischen Notfällen während Ihres Aufenthaltes in der OBERWAID erreichen Sie 24h die Pflege unter der internen Nummer 721.

17. Glossar (erklärungsbedürftige Begriffe)

- Was heisst „randomisiert“?
  Bei vielen Studien werden zwei oder mehrere unterschiedliche Arten der Behandlung verglichen. Zum Beispiel vergleicht man ein echtes Medikament mit einem Scheinmedikament. Man bildet dann zwei Gruppen von Teilnehmenden, die einen bekommen das echte Medikament und die anderen das Scheinmedikament. „Randomisieren“ bedeutet dann, dass ausgelost wird, wer in welche Gruppe kommt. Es ist bei einem solchen Test also Zufall, ob man das echte Medikament erhält oder das Scheinmedikament.

- Was heisst "einfachblind"?
  Eine Studie zu "verblinden" dient dazu, bessere und genauerer Ergebnisse zu erhalten. Von einer "einfachblinden" Studie spricht man, wenn bei der Studie entweder die Studienteilnehmer oder (wie in dieser Studie) die Forschenden nicht wissen, welche Behandlung ein Teilnehmer erhält. Wer was bekommt, lost eine Person aus, die nicht bei dem Test mitmacht.

OBERWAID
DAS HOTEL. DIE KLINIK.

Einwilligungserklärung

Schriftliche Einwilligungserklärung zur Teilnahme an einem Studienprojekt
Bitte lesen Sie dieses Formular sorgfältig durch. Bitte fragen Sie, wenn Sie etwas nicht verstehen oder wissen möchten. Für die Teilnahme ist Ihre schriftliche Einwilligung notwendig.

**BASEC-Nummer (nach Einreichung):**	2018-01268
**Titel der Studie (wissenschaftlich und Laiensprache)**:	Effekte eines Ausdauertrainings auf die Schlafeffizienz bei Patienten mit Depression – EASED Studie<br><br>(Originaltitel: The acute effect of aerobic exercise on sleep in patients with depression – EASED trial)
**verantwortliche Institution (Sponsor mit Adresse)**:	OBERWAID AG<br>Rorschacher Strasse 311<br>9016 St.Gallen
**Ort der Durchführung**:	OBERWAID AG<br>Rorschacher Strasse 311<br>9016 St.Gallen
**Verantwortliche Prüfärztin am Studienort:**<br>Name und Vorname in Druckbuchstaben:	Dr. med. Hildburg Porschke
**Teilnehmerin/Teilnehmer:**<br>Name und Vorname in Druckbuchstaben:<br>Geburtsdatum:	☐ weiblich ☐ männlich

- Ich wurde vom der unterzeichnenden Prüfperson mündlich und schriftlich über den Zweck, den Ablauf der Studie und über mögliche Vor- und Nachteile sowie über eventuelle Risiken informiert.
- Ich nehme an dieser Studie freiwillig teil und akzeptiere den Inhalt der abgegebenen schriftlichen Information. Ich hatte genügend Zeit, meine Entscheidung zu treffen.
- Meine Fragen im Zusammenhang mit der Teilnahme an dieser Studie sind mir beantwortet worden. Ich behalte die schriftliche Information und erhalte eine Kopie meiner schriftlichen Einwilligungserklärung.
- Ich bin einverstanden, dass die zuständigen Fachleute des Sponsors und der zuständigen Ethikkommission zu Prüf- und Kontrollzwecken in meine unverschlüsselten Daten Einsicht nehmen dürfen, jedoch unter strikter Einhaltung der Vertraulichkeit.
- Bei Studienergebnissen, die direkt meine Gesundheit betreffen, werde ich informiert. Wenn ich das nicht wünsche, informiere ich meine Prüfperson.
- Ich weiss, dass meine gesundheitsbezogenen und persönlichen Daten nur in verschlüsselter Form zu Forschungszwecken für diese Studie weitergegeben werden können (auch ins Ausland).
- Ich kann jederzeit und ohne Angabe von Gründen von der Studienteilnahme zurücktreten. Meine weitere medizinische Behandlung ist unabhängig von der Studienteilnahme immer gewährleistet. Die bis zum Rücktritt erhobenen Daten werden für die Auswertung zur Studie verwendet.
- Die Haftpflichtversicherung der Institution kommt für allfällige Schäden auf.

- Ich bin mir bewusst, dass die in der Teilnehmerinformation genannten Pflichten einzuhalten sind. Im Interesse meiner Gesundheit kann mich die Prüfärztin jederzeit von der Studie ausschliessen.

Ort, Datum	Unterschrift Teilnehmerin/Teilnehmer

Bestätigung der Prüfärztin / Prüfperson: Hiermit bestätige ich, dass ich dieser Teilnehmerin/ diesem Teilnehmer Wesen, Bedeutung und Tragweite der Studie erläutert habe. Ich versichere, alle im Zusammenhang mit dieser Studie stehenden Verpflichtungen gemäss des geltenden Rechts zu erfüllen. Sollte ich zu irgendeinem Zeitpunkt während der Durchführung der Studie von Aspekten erfahren, welche die Bereitschaft der Teilnehmerin/ des Teilnehmers zur Teilnahme an der Studie beeinflussen könnten, werde ich sie/ ihn umgehend darüber informieren.

Ort, Datum	Name und Vorname der Prüfperson in Druckbuchstaben<br><br>Unterschrift der Prüfperson

OBERWAID
DAS HOTEL. DIE KLINIK.

Einwilligungserklärung für Weiterverwendung von Daten

**Teilnehmerin/Teilnehmer:**<br>Name und Vorname in Druckbuchstaben:<br>Geburtsdatum:	☐ weiblich ☐ männlich

Ich erlaube, dass meine Daten aus dieser Studie für die medizinische Forschung weiterverwendet werden dürfen. Die Daten werden in einer Datenbank gespeichert und für zukünftige, noch nicht näher definierte Forschungsprojekte auf unbestimmte Zeitdauer verwendet. Diese Einwilligung gilt unbegrenzt.

Ich habe verstanden, dass die Daten verschlüsselt sind und der Schüssel sicher aufbewahrt wird. Die Daten können im In- und Ausland an andere Datenbanken zur Analyse gesendet werden, wenn diese dieselben Standards wie in der Schweiz einhalten. Alle rechtlichen Vorgaben zum Datenschutz werden eingehalten.

Ich entscheide freiwillig und kann diesen Entscheid zu jedem Zeitpunkt wieder zurücknehmen. Wenn ich zurücktrete, werden meine Daten anonymisiert. Ich informiere lediglich meine Prüfärztin und muss diesen Entscheid nicht begründen.

Normalerweise werden alle Daten gesamthaft ausgewertet und die Ergebnisse zusammenfassend publiziert. Sollte sich ein für meine Gesundheit wichtiges Ergebnis ergeben, ist es möglich, dass ich über meine Prüfärztin kontaktiert werde. Wenn ich das nicht wünsche, teile ich es meiner Prüfärztin mit.

Wenn Ergebnisse aus den Daten kommerzialisiert werden, habe ich keinen Anspruch auf Anteil an der kommerziellen Nutzung.

Ort, Datum	Unterschrift Teilnehmerin/ Teilnehmer

Bestätigung der Prüfärztin / Prüfperson: Hiermit bestätige ich, dass ich dieser Teilnehmerin/ diesem Teilnehmer Wesen, Bedeutung und Tragweite der Weiterverwendung von Daten erläutert habe.

Ort, Datum	Name und Vorname der informierenden der informierenden Prüfperson in Druckbuchstaben<br><br>Unterschrift der der Prüfperson
