# Supplementary material for: The acute effects of aerobic exercise on sleep in patients with depression: study protocol for a randomized controlled trial
Source: Trials. 2019 Jun 13;20:352. doi: 10.1186/s13063-019-3415-3 (PMC6567535; doi:10.1186/s13063-019-3415-3)
Supplement: Supplementary file 3 — Electronic case report form (eCRF). (PDF 504 kb) [file 13063_2019_3415_MOESM3_ESM.pdf]

EASED trial - version 232.71

Printed on 22-11-2018 09:28:32 by Gavin Brupbacher

1. Days 0-2 - Screening

Number	Question	Answers
1.1	Informed consent EASED<br>*Field type*: Radiobutton<br>*Variable name*: Consent_EASED<br>*Field required*: Required<br>*Option group name*: Yes/No	○ Yes<br>○ No
1.2	General consent<br>*Field type*: Radiobutton<br>*Variable name*: Consent_General<br>*Field required*: Required<br>*Option group name*: Yes/No	○ Yes<br>○ No
1.3	Psychosomatic in-patient<br>*Field type*: Radiobutton<br>*Variable name*: PS_Reha<br>*Field required*: Not required<br>*Option group name*: Yes/No	○ Yes<br>○ No
1.4	Age<br>*Field type*: Numeric field<br>*Variable name*: Age<br>*Field required*: Not required<br>*Field min*: 18.00<br>*Field max*: 100.00<br>*Measurement Unit*: years	[ ] years
1.5	Primary diagnosis of depression<br>*Notice shown if field's value is equal to Yes: 'Cave: main diagnosis may NOT be F32.3 or F33.3'*<br>*Field type*: Radiobutton<br>*Variable name*: Prim_Diag_Depr<br>*Field required*: Not required<br>*Option group name*: Yes/No	○ Yes<br>○ No
1.6	Use of hypnotics in last 2 weeks<br>*Field type*: Radiobutton<br>*Variable name*: Hypnotics<br>*Field required*: Not required<br>*Option group name*: Yes/No	○ Yes<br>○ No
1.7	Exercise contraindication<br>*Field type*: Radiobutton<br>*Variable name*: Exercise_contraindication<br>*Field required*: Not required<br>*Option group name*: Yes/No	○ Yes<br>○ No

1.8	Use of beta-blockers (with the exception of Carvedilol & Nebivolol)<br>*Field type*: Radiobutton<br>*Variable name*: Beta_blockers<br>*Field required*: Not required<br>*Option group name*: Yes/No	◯ Yes<br>◯ No
1.9	Use of opioids<br>*Field type*: Radiobutton<br>*Variable name*: Opioids<br>*Field required*: Not required<br>*Option group name*: Yes/No	◯ Yes<br>◯ No
1.10	History of epilepsy<br>*Field type*: Radiobutton<br>*Variable name*: Epilepsy<br>*Field required*: Not required<br>*Option group name*: Yes/No	◯ Yes<br>◯ No
1.11	RLS screening questionnaire score<br>*Field type*: Calculation<br>*Variable name*: RLSSQ_score<br>*Field required*: Not required<br>*Field min*: 0.00<br>*Field max*: 10.00	
1.12	Body mass index<br>*Field type*: Numeric field<br>*Variable name*: BMI<br>*Field required*: Not required<br>*Field min*: 0.00<br>*Field max*: 50.00	[ ]
1.13	Preliminary inclusion, pending oxygen desaturation index<br>*Exclude patient if field's value is equal to Exclusion with message: 'The patient is excluded form the study'*<br>*Field type*: Calculation<br>*Variable name*: Preliminary_inclusion<br>*Field required*: Not required<br>*Field min*: 0.00<br>*Field max*: 50.00	
1.13.1	***If 'Preliminary inclusion, pending oxygen desaturation index' is equal to 'Preliminary inclusion' answer this question:***<br>Oxygen desaturation index, baseline<br>*Field type*: Calculation<br>*Variable name*: ODI_screening<br>*Field required*: Not required<br>*Field min*: 0.00<br>*Field max*: 50.00	

1.13.2 ***If 'Preliminary inclusion, pending oxygen desaturation index' is equal to 'Preliminary inclusion' answer this question:***

Definitive inclusion
Exclude patient if field's value is equal to Exclusion with message: 'This patient is excluded from the study'
Field type: Calculation
Variable name: Definitive_inclusion
Field required: Not required
Field min: 0.00
Field max: 50.00

2. Days 0-2 - Graded exercise test

Number	Question	Answers
2.1	Polar watch used for test<br>SM06: forschung@oberwaid.ch & Tatc93kW<br><br>SM07: gavin.brupbacher@oberwaid.ch & Loka75zY<br><br>V800 (Oberwaid) : sport@oberwaid.ch & OberwaidSport<br><br>*Field type*: Dropdown<br>*Variable name*: Polar_test<br>*Field required*: Required<br>*Field min*: 0.00<br>*Field max*: 220.00<br>*Measurement Unit*: beats per min<br>*Option group name*: Polar watch name	○ SM06<br>○ SM07<br>○ V800 Oberwaid
2.2	Watt at 80% of individual anaerobic threshhold<br>*Field type*: Numeric field<br>*Variable name*: Watt_80IAS<br>*Field required*: Required<br>*Field min*: 0.00<br>*Field max*: 400.00<br>*Measurement Unit*: Watt	[ ] Watt
2.3	Expected heart rate at 80% IAS<br>*Field type*: Numeric field<br>*Variable name*: Expected_HR_80IAS<br>*Field required*: Required<br>*Field min*: 40.00<br>*Field max*: 220.00<br>*Measurement Unit*: beats per min	[ ] beats per min
2.4	Expected rate of perceived exertion at 80% IAS<br>*Field type*: Numeric field<br>*Variable name*: Expected_RPE_80IAS<br>*Field required*: Required<br>*Field min*: 6.00<br>*Field max*: 20.00	[ ]
2.5	Test_termination<br>*Field type*: Radiobutton<br>*Variable name*: Test_termination<br>*Field required*: Not required<br>*Field min*: 0.00<br>*Field max*: 400.00<br>*Measurement Unit*: Watt<br>*Option group name*: Yes/No	○ Yes<br>○ No
2.5.1	***If 'Test_termination' is equal to 'Yes' answer this question:***<br>Reason for test termination<br>*Field type*: Multiline Textfield<br>*Variable name*: Test_termination_reason<br>*Field required*: Not required	[ ]

3. Day 3 - Background variables

Number	Question	Answers
3.1	Sex<br>*Field type*: Calculation<br>*Variable name*: Sex<br>*Field required*: Not required	
3.2	Smoker status<br>*Field type*: Calculation<br>*Variable name*: Smoker_status<br>*Field required*: Not required	
3.2.1	***If 'Smoker status' is equal to '1' answer this question:***<br>Packyears of those who stopped smoking<br>*Field type*: Calculation<br>*Variable name*: Packyears_previous<br>*Field required*: Not required<br>*Measurement Unit*: packyears	
3.2.2	***If 'Smoker status' is equal to '2' answer this question:***<br>Packyears of current smokers<br>*Field type*: Calculation<br>*Variable name*: Packyears_current_1<br>*Field required*: Not required<br>*Measurement Unit*: packyears	
3.3	Alcohol consumption<br>*Field type*: Calculation<br>*Variable name*: Alcohol<br>*Field required*: Not required	
3.4	Credibility<br>*Field type*: Calculation<br>*Variable name*: Credibility<br>*Field required*: Not required	
3.5	Expectancy<br>*Field type*: Calculation<br>*Variable name*: Expectancy<br>*Field required*: Not required	

4. Day 3 - PHQ-9

Number	Question	Answers
4.1	Patient Health Questionnaire (PHQ-9) score<br>*Field type*: Calculation<br>*Variable name*: PHQ9<br>*Field required*: Not required<br>*Field min*: 0.00<br>*Field max*: 27.00	

5. Day 3 - HADS

Number	Question	Answers
5.1	Hospital Anxiety & Depression Questionnaire (HADS) depression score<br>*Field type*: Calculation<br>*Variable name*: HADS_depression<br>*Field required*: Not required<br>*Field min*: 0.00<br>*Field max*: 21.00	
5.2	Hospital Anxiety & Depression Questionnaire (HADS) anxiety score<br>*Field type*: Calculation<br>*Variable name*: HADS_anxiety<br>*Field required*: Not required<br>*Field min*: 0.00<br>*Field max*: 21.00	
5.3	Hospital Anxiety & Depression Questionnaire (HADS) sum score<br>*Field type*: Calculation<br>*Variable name*: HADS_sum<br>*Field required*: Not required<br>*Field min*: 0.00<br>*Field max*: 21.00	

6. Day 3 - PSS-10

Number	Question	Answers
6.1	Perceived Stress Scale (PSS-10)<br>*Field type*: Calculation<br>*Variable name*: PSS10<br>*Field required*: Not required<br>*Field min*: 0.00<br>*Field max*: 40.00	

7. Day 3 - PHQ-15

Number	Question	Answers
7.1	Patient Health Questionnaire (PHQ-15)<br>*Field type*: Calculation<br>*Variable name*: PHQ15<br>*Field required*: Not required<br>*Field min*: 0.00<br>*Field max*: 30.00	

8. Day 3 - CIRS

Number	Question	Answers
8.1	Cumulative Illness Rating Scale (CIRS)<br>*Field type*: Calculation<br>*Variable name*: CIRS<br>*Field required*: Not required<br>*Field min*: 0.00<br>*Field max*: 56.00	

9. Day 3 - ESS

Number	Question	Answers
9.1	Epworth Sleepiness Scale (ESS)<br>*Field type*: Calculation<br>*Variable name*: ESS<br>*Field required*: Not required<br>*Field min*: 0.00<br>*Field max*: 24.00	

10. Day 3 - PSQI

Number	Question	Answers
10.1	PSQI component 1: subjective sleep quality<br>*Field type*: Calculation<br>*Variable name*: PSQI_comp_1<br>*Field required*: Not required	
10.2	PSQI component 2: sleep latency<br>*Field type*: Calculation<br>*Variable name*: PSQI_comp_2<br>*Field required*: Not required	
10.3	PSQI component 3: sleep duration<br>*Field type*: Calculation<br>*Variable name*: PSQI_comp_3<br>*Field required*: Not required	
10.4	PSQI component 4 calculation<br>*Field type*: Calculation<br>*Variable name*: PSQI_comp_4calc<br>*Field required*: Not required	
10.5	PSQI component 4: sleep efficacy<br>*Field type*: Calculation<br>*Variable name*: PSQI_comp_4<br>*Field required*: Not required	
10.6	PSQI component 5 calculation<br>*Field type*: Calculation<br>*Variable name*: PSQI_comp_5calc<br>*Field required*: Not required	
10.7	PSQI component 5: sleep disturbances<br>*Field type*: Calculation<br>*Variable name*: PSQI_comp_5<br>*Field required*: Not required	
10.8	PSQI component 6: use of sleeping medication<br>*Field type*: Calculation<br>*Variable name*: PSQI_comp_6<br>*Field required*: Not required	
10.9	PSQI component 7: daytime dysfunction<br>*Field type*: Calculation<br>*Variable name*: PSQI_comp_7<br>*Field required*: Not required	
10.10	Pittsburgh Sleep Quality index (PSQI)<br>*Field type*: Calculation<br>*Variable name*: PSQI<br>*Field required*: Not required<br>*Field min*: 0.00<br>*Field max*: 21.00	

11. Day 3 - DBAS-16

Number	Question	Answers
11.1	Dysfunctional beliefs & attitudes about sleep scale (DBAS-16) consequences<br>*Field type*: Calculation<br>*Variable name*: DBAS16_cons<br>*Field required*: Not required<br>*Field min*: 0.00<br>*Field max*: 10.00	
11.2	Dysfunctional beliefs & attitudes about sleep scale (DBAS-16) worry<br>*Field type*: Calculation<br>*Variable name*: DBAS16_worry<br>*Field required*: Not required<br>*Field min*: 0.00<br>*Field max*: 10.00	
11.3	Dysfunctional beliefs & attitudes about sleep scale (DBAS-16) expectations<br>*Field type*: Calculation<br>*Variable name*: DBAS16_expect<br>*Field required*: Not required<br>*Field min*: 0.00<br>*Field max*: 10.00	
11.4	Dysfunctional beliefs & attitudes about sleep scale (DBAS-16) medication<br>*Field type*: Calculation<br>*Variable name*: DBAS16_med<br>*Field required*: Not required<br>*Field min*: 0.00<br>*Field max*: 10.00	
11.5	Dysfunctional beliefs & attitudes about sleep scale (DBAS-16) sum score<br>*Field type*: Calculation<br>*Variable name*: DBAS16_sum<br>*Field required*: Not required<br>*Field min*: 0.00<br>*Field max*: 10.00	

12. Day 3 - MEQ

Number	Question	Answers
12.1	Morningness-Eveningness Questionnaire (MEQ)<br>*Field type*: Calculation<br>*Variable name*: MEQ<br>*Field required*: Not required<br>*Field min*: 16.00<br>*Field max*: 86.00	

13. Day 3 - FIRST

Number	Question	Answers
13.1	Ford Insomnia Response to Stress sum score<br>*Field type*: Calculation<br>*Variable name*: FIRST_score<br>*Field required*: Not required	

14. Day 3 - Blood pressure

Number	Question	Answers
14.1	Systolic blood pressure day 3, measurement 1<br>*Field type*: Numeric field<br>*Variable name*: Syst_BP_day_3_1<br>*Field required*: Required<br>*Field min*: 50.00<br>*Field max*: 240.00<br>*Measurement Unit*: mmHg	mmHg
14.2	Diastolic blood pressure day 3, measurement 1<br>*Field type*: Numeric field<br>*Variable name*: Dia_BP_day_3_1<br>*Field required*: Required<br>*Field min*: 20.00<br>*Field max*: 170.00<br>*Measurement Unit*: mmHg	mmHg
14.3	Systolic blood pressure day 3, measurement 2<br>*Field type*: Numeric field<br>*Variable name*: Syst_BP_day_3_2<br>*Field required*: Required<br>*Field min*: 50.00<br>*Field max*: 240.00<br>*Measurement Unit*: mmHg	mmHg
14.4	Diastolic blood pressure day 3, measurement 2<br>*Field type*: Numeric field<br>*Variable name*: Dia_BP_day_3_2<br>*Field required*: Required<br>*Field min*: 20.00<br>*Field max*: 170.00<br>*Measurement Unit*: mmHg	mmHg
14.5	Systolic blood pressure day 3, average of measurement 1 & 2<br>*Field type*: Calculation<br>*Variable name*: Syst_BP_day_3<br>*Field required*: Not required<br>*Field min*: 50.00<br>*Field max*: 240.00<br>*Measurement Unit*: mmHg	
14.6	Diastolic blood pressure day 3, average of measurement 1 & 2<br>*Field type*: Calculation<br>*Variable name*: Dia_BP_day_3<br>*Field required*: Not required<br>*Field min*: 20.00<br>*Field max*: 170.00<br>*Measurement Unit*: mmHg	

14.7 Diastolic blood pressure day 3
Field type: Calculation
Variable name: MAP_day_3
Field required: Not required
Field min: 20.00
Field max: 170.00
Measurement Unit: mmHg

15. Baseline - Polysomnography, sleep assessement 1

Number	Question	Answers
15.1	Date of baseline polysomnography<br>*Field type*: Date<br>*Variable name*: Date_PSG_1<br>*Field required*: Required<br>*Measurement Unit*: min	[ ] [ ] [ ] *(dd-mm-yyyy)*
15.2	Sleep stage artefact duration<br>*Field type*: Numeric field<br>*Variable name*: Sleep_artefact_min_1<br>*Field required*: Required<br>*Measurement Unit*: min	[ ] min
15.3	Sleep stage artefact % total recording time<br>*Field type*: Numeric field<br>*Variable name*: Sleep_artefact_TRT_1<br>*Field required*: Required<br>*Measurement Unit*: %	[ ] %
15.4	Total recording time (TRT) night 1<br>*Field type*: Numeric field<br>*Variable name*: TRT_1<br>*Field required*: Required<br>*Measurement Unit*: min	[ ] min
15.5	Total sleep time (TST) night 1<br>*Field type*: Numeric field<br>*Variable name*: TST_1<br>*Field required*: Required<br>*Measurement Unit*: min	[ ] min
15.6	Sleep onset latency (SOL) night 1<br>*Field type*: Numeric field<br>*Variable name*: SOL_1<br>*Field required*: Required<br>*Measurement Unit*: min	[ ] min
15.7	Wake after sleep onset (WASO) night 1<br>*Field type*: Numeric field<br>*Variable name*: WASO_1<br>*Field required*: Required<br>*Measurement Unit*: min	[ ] min
15.8	Number of awakenings (NA) night 1<br>*Field type*: Numeric field<br>*Variable name*: NA_1<br>*Field required*: Required	[ ]
15.9	Sleep efficiency 1 (SE) night 1<br>*Field type*: Numeric field<br>*Variable name*: SE_1<br>*Field required*: Required<br>*Field min*: 0.00<br>*Field max*: 100.00<br>*Measurement Unit*: %	[ ] %

15.10	Wake min night 1<br>*Field type*: Numeric field<br>*Variable name*: Wake_min_1<br>*Field required*: Required<br>*Measurement Unit*: min	[ ]	min
15.11	Wake %TRT night 1<br>*Field type*: Numeric field<br>*Variable name*: Wake_TRT_1<br>*Field required*: Required<br>*Field min*: 0.00<br>*Field max*: 100.00<br>*Measurement Unit*: %	[ ]	%
15.12	N1 min night 1<br>*Field type*: Numeric field<br>*Variable name*: N1_min_1<br>*Field required*: Required<br>*Measurement Unit*: min	[ ]	min
15.13	N1 %TRT night 1<br>*Field type*: Numeric field<br>*Variable name*: N1_TRT_1<br>*Field required*: Required<br>*Field min*: 0.00<br>*Field max*: 100.00<br>*Measurement Unit*: %	[ ]	%
15.14	N1 %TST night 1<br>*Field type*: Calculation<br>*Variable name*: N1_TST_1<br>*Field required*: Not required<br>*Field min*: 0.00<br>*Field max*: 100.00<br>*Measurement Unit*: %		
15.15	N2 min night 1<br>*Field type*: Numeric field<br>*Variable name*: N2_min_1<br>*Field required*: Required<br>*Measurement Unit*: min	[ ]	min
15.16	N2 %TRT night 1<br>*Field type*: Numeric field<br>*Variable name*: N2_TRT_1<br>*Field required*: Required<br>*Field min*: 0.00<br>*Field max*: 100.00<br>*Measurement Unit*: %	[ ]	%
15.17	N2 %TST night 1<br>*Field type*: Calculation<br>*Variable name*: N2_TST_1<br>*Field required*: Not required<br>*Field min*: 0.00<br>*Field max*: 100.00<br>*Measurement Unit*: %		

15.18 N3 min night 1
Field type: Numeric field
Variable name: N3_min_1
Field required: Required
Measurement Unit: min

[ ] min

15.19 N3 %TRT night 1
Field type: Numeric field
Variable name: N3_TRT_1
Field required: Required
Field min: 0.00
Field max: 100.00
Measurement Unit: %

[ ] %

15.20 N3 %total sleep time night 1
Field type: Calculation
Variable name: N3_TST_1
Field required: Not required
Field min: 0.00
Field max: 100.00
Measurement Unit: %

15.21 Light Sleep min night 1
Field type: Calculation
Variable name: LS_min_1
Field required: Not required
Measurement Unit: min

15.22 Light Sleep %TST night 1
Field type: Calculation
Variable name: LS_TST_1
Field required: Not required
Field min: 0.00
Field max: 100.00
Measurement Unit: %

15.23 Slow wave sleep (SWS) min night 1
Field type: Numeric field
Variable name: SWS_min_1
Field required: Required
Measurement Unit: min

[ ] min

15.24 Slow wave sleep (SWS) %TRT night 1
Field type: Numeric field
Variable name: SWS_TRT_1
Field required: Required
Field min: 0.00
Field max: 100.00
Measurement Unit: %

[ ] %

15.25 Slow wave sleep (SWS) %TST night 1
Field type: Calculation
Variable name: SWS_TST_1
Field required: Not required
Field min: 0.00
Field max: 100.00
Measurement Unit: %

15.26	NREM (non-REM) sleep min night 1<br>*Field type*: Numeric field<br>*Variable name*: NREM_min_1<br>*Field required*: Required<br>*Measurement Unit*: min	[ ]	min
15.27	NREM (non-REM) sleep %TRT night 1<br>*Field type*: Numeric field<br>*Variable name*: NREM_TRT_1<br>*Field required*: Required<br>*Field min*: 0.00<br>*Field max*: 100.00<br>*Measurement Unit*: %	[ ]	%
15.28	NREM (non-REM) sleep %TST night 1<br>*Field type*: Calculation<br>*Variable name*: NREM_TST_1<br>*Field required*: Not required<br>*Field min*: 0.00<br>*Field max*: 100.00<br>*Measurement Unit*: %		
15.29	REM min night 1<br>*Field type*: Numeric field<br>*Variable name*: REM_min_1<br>*Field required*: Required<br>*Measurement Unit*: min	[ ]	min
15.30	REM % TRT night 1<br>*Field type*: Numeric field<br>*Variable name*: REM_TRT_1<br>*Field required*: Required<br>*Field min*: 0.00<br>*Field max*: 100.00<br>*Measurement Unit*: %	[ ]	%
15.31	REM % total sleep time night 1<br>*Field type*: Calculation<br>*Variable name*: REM_TST_1<br>*Field required*: Not required<br>*Field min*: 0.00<br>*Field max*: 100.00<br>*Measurement Unit*: %		
15.32	REMLAT min night 1<br>*Field type*: Numeric field<br>*Variable name*: REMLAT_min_1<br>*Field required*: Required<br>*Measurement Unit*: min	[ ]	min
15.33	Stage shift index night 1<br>*Field type*: Numeric field<br>*Variable name*: SSI_1<br>*Field required*: Required	[ ]	
15.34	Oxygen saturation artefact duration during sleep night 1<br>*Field type*: Numeric field<br>*Variable name*: SPO2_artefact_1<br>*Field required*: Required<br>*Measurement Unit*: min	[ ]	min

15.35	Oxygen desaturation index from night 1<br>*Field type*: Numeric field<br>*Variable name*: ODI_1<br>*Field required*: Required	
15.36	Pulse transit time artefact duration during sleep night 1<br>*Field type*: Numeric field<br>*Variable name*: PTT_sleep_artefact_min_1<br>*Field required*: Required<br>*Measurement Unit*: min	min
15.37	Pulse transit time artefact duration during REM night 1<br>*Field type*: Numeric field<br>*Variable name*: PTT_REM_artefact_min_1<br>*Field required*: Required<br>*Measurement Unit*: min	min
15.38	Pulse transit time artefact duration during non-REM night 1<br>*Field type*: Numeric field<br>*Variable name*: PTT_NREM_artefact_min_1<br>*Field required*: Required<br>*Measurement Unit*: min	min
15.39	TST mean systolic BP night 1<br>*Field type*: Numeric field<br>*Variable name*: TST_Sys_BP_1<br>*Field required*: Required<br>*Measurement Unit*: mmHg	mmHg
15.40	TST mean diastolic BP night 1<br>*Field type*: Numeric field<br>*Variable name*: TST_Dia_BP_1<br>*Field required*: Required<br>*Measurement Unit*: mmHg	mmHg
15.41	NREM mean systolic BP night 1<br>*Field type*: Numeric field<br>*Variable name*: NREM_Sys_BP_1<br>*Field required*: Required<br>*Measurement Unit*: mmHg	mmHg
15.42	NREM mean diastolic BP night 1<br>*Field type*: Numeric field<br>*Variable name*: NREM_Dia_BP_1<br>*Field required*: Required<br>*Measurement Unit*: mmHg	mmHg
15.43	REM mean systolic BP night 1<br>*Field type*: Numeric field<br>*Variable name*: REM_Sys_BP_1<br>*Field required*: Required<br>*Measurement Unit*: mmHg	mmHg
15.44	REM mean diastolic BP night 1<br>*Field type*: Numeric field<br>*Variable name*: REM_Dia_BP_1<br>*Field required*: Required<br>*Measurement Unit*: mmHg	mmHg

15.45 Mean arterial pressure night 1
Field type: Calculation
Variable name: MAP_night_1
Field required: Not required

16. Baseline - Pre sleep HRV, sleep assessment 1

Number	Question	Answers
16.1	Start and stop time of pre-sleep segment, night 1<br>*Field type*: Textfield<br>*Variable name*: Time_PS_1<br>*Field required*: Required<br>*Measurement Unit*: hh:mm:ss-hh:mm:ss	[ ] hh:mm:ss-hh:mm:ss
16.2	Artefact of pre-sleep segment, night 1<br>*Field type*: Numeric field<br>*Variable name*: Artefact_PS_1<br>*Field required*: Required<br>*Field min*: 0.00<br>*Field max*: 100.00<br>*Measurement Unit*: %	[ ] %
16.3	Mean heart rate, Pre-sleep, night 1<br>*Field type*: Numeric field<br>*Variable name*: meanHR_PS_1<br>*Field required*: Required<br>*Measurement Unit*: beats per min	[ ] beats per min
16.4	RMSSD Pre-sleep HRV night 1<br>*Field type*: Numeric field<br>*Variable name*: RMSSD_PS_1<br>*Field required*: Required<br>*Measurement Unit*: ms	[ ] ms
16.5	SDNN Pre-sleep HRV night 1<br>*Field type*: Numeric field<br>*Variable name*: SDNN_PS_1<br>*Field required*: Required<br>*Measurement Unit*: ms	[ ] ms
16.6	Total power, based on Lomb Scargle Periodogram, Pre-sleep, night 1<br>*Field type*: Numeric field<br>*Variable name*: TP_LSP_PS_1<br>*Field required*: Required<br>*Measurement Unit*: Hz	[ ] Hz
16.7	Low frequency power, based on Lomb Scargle Periodogram, Pre-sleep, night 1<br>*Field type*: Numeric field<br>*Variable name*: LF_LSP_PS_1<br>*Field required*: Required<br>*Measurement Unit*: Hz	[ ] Hz
16.8	High frequency power, based on Lomb Scargle Periodogram, Pre-sleep, night 1<br>*Field type*: Numeric field<br>*Variable name*: HF_LSP_PS_1<br>*Field required*: Required<br>*Measurement Unit*: Hz	[ ] Hz

16.9	LF/HF, based on Lomb Scargle Periodogram, Pre-sleep, night 1<br>*Field type*: Numeric field<br>*Variable name*: LFHF_LSP_PS_1<br>*Field required*: Required	
16.10	Total power, based on Fast Fourier Transformation, Pre-sleep night 1<br>*Field type*: Numeric field<br>*Variable name*: TP_FFT_PS_1<br>*Field required*: Required<br>*Measurement Unit*: Hz	Hz
16.11	Low frequency power, based on Fast Fourier Transformation, Pre-sleep night 1<br>*Field type*: Numeric field<br>*Variable name*: LF_FFT_PS_1<br>*Field required*: Required<br>*Measurement Unit*: Hz	Hz
16.12	High frequency power, based on Fast Fourier Transformation, Pre-sleep night 1<br>*Field type*: Numeric field<br>*Variable name*: HF_FFT_PS_1<br>*Field required*: Required<br>*Measurement Unit*: Hz	Hz
16.13	LF/HF, based on Fast Fourier Transformation, Pre-sleep night 1<br>*Field type*: Numeric field<br>*Variable name*: LFHF_FFT_PS_1<br>*Field required*: Required	

17. Baseline - Nocturnal hour 1 HRV, sleep assessment 1

Number	Question	Answers
17.1	Start and stop time of nocturnal hour 1 segment, night 1<br>*Field type*: Textfield<br>*Variable name*: Time_h1_1<br>*Field required*: Required<br>*Measurement Unit*: hh:mm:ss-hh:mm:ss	[ ] hh:mm:ss-hh:mm:ss
17.2	Artefact of nocturnal hour 1 segment, night 1<br>*Field type*: Numeric field<br>*Variable name*: Artefact_h1_1<br>*Field required*: Required<br>*Field min*: 0.00<br>*Field max*: 100.00<br>*Measurement Unit*: %	[ ] %
17.3	Mean heart rate, nocturnal hour 1, night 1<br>*Field type*: Numeric field<br>*Variable name*: meanHR_h1_1<br>*Field required*: Required<br>*Measurement Unit*: beats per min	[ ] beats per min
17.4	RMSSD, Nocturnal hour 1, night 1<br>*Field type*: Numeric field<br>*Variable name*: RMSSD_h1_1<br>*Field required*: Required<br>*Measurement Unit*: ms	[ ] ms
17.5	SDNN, Nocturnal hour 1, night 1<br>*Field type*: Numeric field<br>*Variable name*: SDNN_h1_1<br>*Field required*: Required<br>*Measurement Unit*: ms	[ ] ms
17.6	SDANN, Nocturnal hour 1, night 1<br>*Field type*: Numeric field<br>*Variable name*: SDANN_h1_1<br>*Field required*: Required<br>*Measurement Unit*: ms	[ ] ms
17.7	Total power, based on Lomb Scargle Periodogram, Nocturnal hour 1, night 1<br>*Field type*: Numeric field<br>*Variable name*: TP_LSP_h1_1<br>*Field required*: Required<br>*Measurement Unit*: Hz	[ ] Hz
17.8	Low frequency power, based on Lomb Scargle Periodogram, Nocturnal hour 1, night 1<br>*Field type*: Numeric field<br>*Variable name*: LF_LSP_h1_1<br>*Field required*: Required<br>*Measurement Unit*: Hz	[ ] Hz

17.9	High frequency power, based on Lomb Scargle Periodogram, Nocturnal hour 1, night 1<br>*Field type*: Numeric field<br>*Variable name*: HF_LSP_h1_1<br>*Field required*: Required<br>*Measurement Unit*: Hz	Hz
17.10	LF/HF, based on Lomb Scargle Periodogram, Nocturnal hour 1, night 1<br>*Field type*: Numeric field<br>*Variable name*: LFHF_LSP_h1_1<br>*Field required*: Required	
17.11	Total power, based on Fast Fourier Transformation, Nocturnal hour 1, night 1<br>*Field type*: Numeric field<br>*Variable name*: TP_FFT_h1_1<br>*Field required*: Required<br>*Measurement Unit*: Hz	Hz
17.12	Low frequency power, based on Fast Fourier Transformation, Nocturnal hour 1, night 1<br>*Field type*: Numeric field<br>*Variable name*: LF_FFT_h1_1<br>*Field required*: Required<br>*Measurement Unit*: Hz	Hz
17.13	High frequency power, based on Fast Fourier Transformation, Nocturnal hour 1, night 1<br>*Field type*: Numeric field<br>*Variable name*: HF_FFT_h1_1<br>*Field required*: Required<br>*Measurement Unit*: Hz	Hz
17.14	LF/HF, based on Fast Fourier Transformation, Nocturnal hour 1, night 1<br>*Field type*: Numeric field<br>*Variable name*: LFHF_FFT_h1_1<br>*Field required*: Required	

18. Baseline - Nocturnal hour 2 HRV, sleep assessment 1

Number	Question	Answers
18.1	Start and stop time of nocturnal hour 2 segment, night 1<br>*Field type*: Textfield<br>*Variable name*: Time_h2_1<br>*Field required*: Required<br>*Measurement Unit*: hh:mm:ss-hh:mm:ss	[ ] hh:mm:ss-hh:mm:ss
18.2	Artefact of nocturnal hour 2 segment, night 1<br>*Field type*: Numeric field<br>*Variable name*: Artefact_h2_1<br>*Field required*: Required<br>*Field min*: 0.00<br>*Field max*: 100.00<br>*Measurement Unit*: %	[ ] %
18.3	Mean heart rate, nocturnal hour 2, night 1<br>*Field type*: Numeric field<br>*Variable name*: meanHR_h2_1<br>*Field required*: Required<br>*Measurement Unit*: beats per min	[ ] beats per min
18.4	RMSSD, Nocturnal hour 2, night 1<br>*Field type*: Numeric field<br>*Variable name*: RMSSD_h2_1<br>*Field required*: Required<br>*Measurement Unit*: ms	[ ] ms
18.5	SDNN, Nocturnal hour 2, night 1<br>*Field type*: Numeric field<br>*Variable name*: SDNN_h2_1<br>*Field required*: Required<br>*Measurement Unit*: ms	[ ] ms
18.6	SDANN, Nocturnal hour 2, night 1<br>*Field type*: Numeric field<br>*Variable name*: SDANN_h2_1<br>*Field required*: Required<br>*Measurement Unit*: ms	[ ] ms
18.7	Total power, based on Lomb Scargle Periodogram, Nocturnal hour 2, night 1<br>*Field type*: Numeric field<br>*Variable name*: TP_LSP_h2_1<br>*Field required*: Required<br>*Measurement Unit*: Hz	[ ] Hz
18.8	Low frequency power, based on Lomb Scargle Periodogram, Nocturnal hour 2, night 1<br>*Field type*: Numeric field<br>*Variable name*: LF_LSP_h2_1<br>*Field required*: Required<br>*Measurement Unit*: Hz	[ ] Hz

18.9	High frequency power, based on Lomb Scargle Periodogram, Nocturnal hour 2, night 1<br>*Field type*: Numeric field<br>*Variable name*: HF_LSP_h2_1<br>*Field required*: Required<br>*Measurement Unit*: Hz	Hz
18.10	LF/HF, based on Lomb Scargle Periodogram, Nocturnal hour 2, night 1<br>*Field type*: Numeric field<br>*Variable name*: LFHF_LSP_h2_1<br>*Field required*: Required	
18.11	Total power, based on Fast Fourier Transformation, Nocturnal hour 2, night 1<br>*Field type*: Numeric field<br>*Variable name*: TP_FFT_h2_1<br>*Field required*: Required<br>*Measurement Unit*: Hz	Hz
18.12	Low frequency power, based on Fast Fourier Transformation, Nocturnal hour 2, night 1<br>*Field type*: Numeric field<br>*Variable name*: LF_FFT_h2_1<br>*Field required*: Required<br>*Measurement Unit*: Hz	Hz
18.13	High frequency power, based on Fast Fourier Transformation, Nocturnal hour 2, night 1<br>*Field type*: Numeric field<br>*Variable name*: HF_FFT_h2_1<br>*Field required*: Required<br>*Measurement Unit*: Hz	Hz
18.14	LF/HF, based on Fast Fourier Transformation, Nocturnal hour 2, night 1<br>*Field type*: Numeric field<br>*Variable name*: LFHF_FFT_h2_1<br>*Field required*: Required	

19. Baseline - Nocturnal hour 3 HRV, sleep assessment 1

Number	Question	Answers
19.1	Start and stop time of nocturnal hour 3 segment, night 1<br>*Field type*: Textfield<br>*Variable name*: Time_h3_1<br>*Field required*: Required<br>*Measurement Unit*: hh:mm:ss-hh:mm:ss	hh:mm:ss-hh:mm:ss
19.2	Artefact of nocturnal hour 3 segment, night 1<br>*Field type*: Numeric field<br>*Variable name*: Artefact_h3_1<br>*Field required*: Required<br>*Field min*: 0.00<br>*Field max*: 100.00<br>*Measurement Unit*: %	%
19.3	Mean heart rate, nocturnal hour 3, night 1<br>*Field type*: Numeric field<br>*Variable name*: meanHR_h3_1<br>*Field required*: Required<br>*Measurement Unit*: beats per min	beats per min
19.4	RMSSD, Nocturnal hour 3, night 1<br>*Field type*: Numeric field<br>*Variable name*: RMSSD_h3_1<br>*Field required*: Required<br>*Measurement Unit*: ms	ms
19.5	SDNN, Nocturnal hour 3, night 1<br>*Field type*: Numeric field<br>*Variable name*: SDNN_h3_1<br>*Field required*: Required<br>*Measurement Unit*: ms	ms
19.6	SDANN, Nocturnal hour 3, night 1<br>*Field type*: Numeric field<br>*Variable name*: SDANN_h3_1<br>*Field required*: Required<br>*Measurement Unit*: ms	ms
19.7	Total power, based on Lomb Scargle Periodogram, Nocturnal hour 3, night 1<br>*Field type*: Numeric field<br>*Variable name*: TP_LSP_h3_1<br>*Field required*: Required<br>*Measurement Unit*: Hz	Hz
19.8	Low frequency power, based on Lomb Scargle Periodogram, Nocturnal hour 3, night 1<br>*Field type*: Numeric field<br>*Variable name*: LF_LSP_h3_1<br>*Field required*: Required<br>*Measurement Unit*: Hz	Hz

19.9	High frequency power, based on Lomb Scargle Periodogram, Nocturnal hour 3, night 1<br>*Field type*: Numeric field<br>*Variable name*: HF_LSP_h3_1<br>*Field required*: Required<br>*Measurement Unit*: Hz	Hz
19.10	LF/HF, based on Lomb Scargle Periodogram, Nocturnal hour 3, night 1<br>*Field type*: Numeric field<br>*Variable name*: LFHF_LSP_h3_1<br>*Field required*: Required	
19.11	Total power, based on Fast Fourier Transformation, Nocturnal hour 3, night 1<br>*Field type*: Numeric field<br>*Variable name*: TP_FFT_h3_1<br>*Field required*: Required<br>*Measurement Unit*: Hz	Hz
19.12	Low frequency power, based on Fast Fourier Transformation, Nocturnal hour 3, night 1<br>*Field type*: Numeric field<br>*Variable name*: LF_FFT_h3_1<br>*Field required*: Required<br>*Measurement Unit*: Hz	Hz
19.13	High frequency power, based on Fast Fourier Transformation, Nocturnal hour 3, night 1<br>*Field type*: Numeric field<br>*Variable name*: HF_FFT_h3_1<br>*Field required*: Required<br>*Measurement Unit*: Hz	Hz
19.14	LF/HF, based on Fast Fourier Transformation, Nocturnal hour 3, night 1<br>*Field type*: Numeric field<br>*Variable name*: LFHF_FFT_h3_1<br>*Field required*: Required	

20. Baseline - Nocturnal hour 4 HRV, sleep assessment 1

Number	Question	Answers
20.1	Start and stop time of nocturnal hour 4 segment, night 1<br>*Field type*: Textfield<br>*Variable name*: Time_h4_1<br>*Field required*: Required<br>*Measurement Unit*: hh:mm:ss-hh:mm:ss	[ ] hh:mm:ss-hh:mm:ss
20.2	Artefact of nocturnal hour 4 segment, night 1<br>*Field type*: Numeric field<br>*Variable name*: Artefact_h4_1<br>*Field required*: Required<br>*Field min*: 0.00<br>*Field max*: 100.00<br>*Measurement Unit*: %	[ ] %
20.3	Mean heart rate, nocturnal hour 4, night 1<br>*Field type*: Numeric field<br>*Variable name*: meanHR_h4_1<br>*Field required*: Required<br>*Measurement Unit*: beats per min	[ ] beats per min
20.4	RMSSD, Nocturnal hour 4, night 1<br>*Field type*: Numeric field<br>*Variable name*: RMSSD_h4_1<br>*Field required*: Required<br>*Measurement Unit*: ms	[ ] ms
20.5	SDNN, Nocturnal hour 4, night 1<br>*Field type*: Numeric field<br>*Variable name*: SDNN_h4_1<br>*Field required*: Required<br>*Measurement Unit*: ms	[ ] ms
20.6	SDANN, Nocturnal hour 4, night 1<br>*Field type*: Numeric field<br>*Variable name*: SDANN_h4_1<br>*Field required*: Required<br>*Measurement Unit*: ms	[ ] ms
20.7	Total power, based on Lomb Scargle Periodogram, Nocturnal hour 4, night 1<br>*Field type*: Numeric field<br>*Variable name*: TP_LSP_h4_1<br>*Field required*: Required<br>*Measurement Unit*: Hz	[ ] Hz
20.8	Low frequency power, based on Lomb Scargle Periodogram, Nocturnal hour 4, night 1<br>*Field type*: Numeric field<br>*Variable name*: LF_LSP_h4_1<br>*Field required*: Required<br>*Measurement Unit*: Hz	[ ] Hz

20.9	High frequency power, based on Lomb Scargle Periodogram, Nocturnal hour 4, night 1<br>*Field type*: Numeric field<br>*Variable name*: HF_LSP_h4_1<br>*Field required*: Required<br>*Measurement Unit*: Hz	Hz
20.10	LF/HF, based on Lomb Scargle Periodogram, Nocturnal hour 4, night 1<br>*Field type*: Numeric field<br>*Variable name*: LFHF_LSP_h4_1<br>*Field required*: Required	
20.11	Total power, based on Fast Fourier Transformation, Nocturnal hour 4, night 1<br>*Field type*: Numeric field<br>*Variable name*: TP_FFT_h4_1<br>*Field required*: Required<br>*Measurement Unit*: Hz	Hz
20.12	Low frequency power, based on Fast Fourier Transformation, Nocturnal hour 4, night 1<br>*Field type*: Numeric field<br>*Variable name*: LF_FFT_h4_1<br>*Field required*: Required<br>*Measurement Unit*: Hz	Hz
20.13	High frequency power, based on Fast Fourier Transformation, Nocturnal hour 4, night 1<br>*Field type*: Numeric field<br>*Variable name*: HF_FFT_h4_1<br>*Field required*: Required<br>*Measurement Unit*: Hz	Hz
20.14	LF/HF, based on Fast Fourier Transformation, Nocturnal hour 4, night 1<br>*Field type*: Numeric field<br>*Variable name*: LFHF_FFT_h4_1<br>*Field required*: Required	

21. Baseline - Nocturnal hour 5 HRV, sleep assessment 1

Number	Question	Answers
21.1	Start and stop time of nocturnal hour 5 segment, night 1<br>*Field type*: Textfield<br>*Variable name*: Time_h5_1<br>*Field required*: Required<br>*Measurement Unit*: hh:mm:ss-hh:mm:ss	[ ] hh:mm:ss-hh:mm:ss
21.2	Artefact of nocturnal hour 5 segment, night 1<br>*Field type*: Numeric field<br>*Variable name*: Artefact_h5_1<br>*Field required*: Required<br>*Field min*: 0.00<br>*Field max*: 100.00<br>*Measurement Unit*: %	[ ] %
21.3	Mean heart rate, nocturnal hour 5, night 1<br>*Field type*: Numeric field<br>*Variable name*: meanHR_h5_1<br>*Field required*: Required<br>*Measurement Unit*: beats per min	[ ] beats per min
21.4	RMSSD, Nocturnal hour 5, night 1<br>*Field type*: Numeric field<br>*Variable name*: RMSSD_h5_1<br>*Field required*: Required<br>*Measurement Unit*: ms	[ ] ms
21.5	SDNN, Nocturnal hour 5, night 1<br>*Field type*: Numeric field<br>*Variable name*: SDNN_h5_1<br>*Field required*: Required<br>*Measurement Unit*: ms	[ ] ms
21.6	SDANN, Nocturnal hour 5, night 1<br>*Field type*: Numeric field<br>*Variable name*: SDANN_h5_1<br>*Field required*: Required<br>*Measurement Unit*: ms	[ ] ms
21.7	Total power, based on Lomb Scargle Periodogram, Nocturnal hour 5, night 1<br>*Field type*: Numeric field<br>*Variable name*: TP_LSP_h5_1<br>*Field required*: Required<br>*Measurement Unit*: Hz	[ ] Hz
21.8	Low frequency power, based on Lomb Scargle Periodogram, Nocturnal hour 5, night 1<br>*Field type*: Numeric field<br>*Variable name*: LF_LSP_h5_1<br>*Field required*: Required<br>*Measurement Unit*: Hz	[ ] Hz

21.9	High frequency power, based on Lomb Scargle Periodogram, Nocturnal hour 5, night 1<br>*Field type*: Numeric field<br>*Variable name*: HF_LSP_h5_1<br>*Field required*: Required<br>*Measurement Unit*: Hz	Hz
21.10	LF/HF, based on Lomb Scargle Periodogram, Nocturnal hour 5, night 1<br>*Field type*: Numeric field<br>*Variable name*: LFHF_LSP_h5_1<br>*Field required*: Required	
21.11	Total power, based on Fast Fourier Transformation, Nocturnal hour 5, night 1<br>*Field type*: Numeric field<br>*Variable name*: TP_FFT_h5_1<br>*Field required*: Required<br>*Measurement Unit*: Hz	Hz
21.12	Low frequency power, based on Fast Fourier Transformation, Nocturnal hour 5, night 1<br>*Field type*: Numeric field<br>*Variable name*: LF_FFT_h5_1<br>*Field required*: Required<br>*Measurement Unit*: Hz	Hz
21.13	High frequency power, based on Fast Fourier Transformation, Nocturnal hour 5, night 1<br>*Field type*: Numeric field<br>*Variable name*: HF_FFT_h5_1<br>*Field required*: Required<br>*Measurement Unit*: Hz	Hz
21.14	LF/HF, based on Fast Fourier Transformation, Nocturnal hour 5, night 1<br>*Field type*: Numeric field<br>*Variable name*: LFHF_FFT_h5_1<br>*Field required*: Required	

22. Baseline - Nocturnal hour 6 HRV, sleep assessment 1

Number	Question	Answers
22.1	Start and stop time of nocturnal hour 6 segment, night 1<br>*Field type*: Textfield<br>*Variable name*: Time_h6_1<br>*Field required*: Required<br>*Measurement Unit*: hh:mm:ss-hh:mm:ss	hh:mm:ss-hh:mm:ss
22.2	Artefact of nocturnal hour 6 segment, night 1<br>*Field type*: Numeric field<br>*Variable name*: Artefact_h6_1<br>*Field required*: Required<br>*Field min*: 0.00<br>*Field max*: 100.00<br>*Measurement Unit*: %	%
22.3	Mean heart rate, nocturnal hour 6, night 1<br>*Field type*: Numeric field<br>*Variable name*: meanHR_h6_1<br>*Field required*: Required<br>*Measurement Unit*: beats per min	beats per min
22.4	RMSSD, Nocturnal hour 6, night 1<br>*Field type*: Numeric field<br>*Variable name*: RMSSD_h6_1<br>*Field required*: Required<br>*Measurement Unit*: ms	ms
22.5	SDNN, Nocturnal hour 6, night 1<br>*Field type*: Numeric field<br>*Variable name*: SDNN_h6_1<br>*Field required*: Required<br>*Measurement Unit*: ms	ms
22.6	SDANN, Nocturnal hour 6, night 1<br>*Field type*: Numeric field<br>*Variable name*: SDANN_h6_1<br>*Field required*: Required<br>*Measurement Unit*: ms	ms
22.7	Total power, based on Lomb Scargle Periodogram, Nocturnal hour 6, night 1<br>*Field type*: Numeric field<br>*Variable name*: TP_LSP_h6_1<br>*Field required*: Required<br>*Measurement Unit*: Hz	Hz
22.8	Low frequency power, based on Lomb Scargle Periodogram, Nocturnal hour 6, night 1<br>*Field type*: Numeric field<br>*Variable name*: LF_LSP_h6_1<br>*Field required*: Required<br>*Measurement Unit*: Hz	Hz

22.9	High frequency power, based on Lomb Scargle Periodogram, Nocturnal hour 6, night 1<br>*Field type*: Numeric field<br>*Variable name*: HF_LSP_h6_1<br>*Field required*: Required<br>*Measurement Unit*: Hz	Hz
22.10	LF/HF, based on Lomb Scargle Periodogram, Nocturnal hour 6, night 1<br>*Field type*: Numeric field<br>*Variable name*: LFHF_LSP_h6_1<br>*Field required*: Required	
22.11	Total power, based on Fast Fourier Transformation, Nocturnal hour 6, night 1<br>*Field type*: Numeric field<br>*Variable name*: TP_FFT_h6_1<br>*Field required*: Required<br>*Measurement Unit*: Hz	Hz
22.12	Low frequency power, based on Fast Fourier Transformation, Nocturnal hour 6, night 1<br>*Field type*: Numeric field<br>*Variable name*: LF_FFT_h6_1<br>*Field required*: Required<br>*Measurement Unit*: Hz	Hz
22.13	High frequency power, based on Fast Fourier Transformation, Nocturnal hour 6, night 1<br>*Field type*: Numeric field<br>*Variable name*: HF_FFT_h6_1<br>*Field required*: Required<br>*Measurement Unit*: Hz	Hz
22.14	LF/HF, based on Fast Fourier Transformation, Nocturnal hour 6, night 1<br>*Field type*: Numeric field<br>*Variable name*: LFHF_FFT_h6_1<br>*Field required*: Required	

23. Baseline - Morning HRV, sleep assessment 1

Number	Question	Answers
23.1	Start and stop time of morning segment, night 1<br>*Field type*: Textfield<br>*Variable name*: Time_m_1<br>*Field required*: Required<br>*Measurement Unit*: hh:mm:ss-hh:mm:ss	hh:mm:ss-hh:mm:ss
23.2	Artefact of morning segment, night 1<br>*Field type*: Numeric field<br>*Variable name*: Artefact_m_1<br>*Field required*: Required<br>*Field min*: 0.00<br>*Field max*: 100.00<br>*Measurement Unit*: %	%
23.3	Mean heart rate, morning, night 1<br>*Field type*: Numeric field<br>*Variable name*: meanHR_m_1<br>*Field required*: Required<br>*Measurement Unit*: beats per min	beats per min
23.4	RMSSD morning HRV night 1<br>*Field type*: Numeric field<br>*Variable name*: RMSSD_m_1<br>*Field required*: Required<br>*Measurement Unit*: ms	ms
23.5	SDNN morning HRV night 1<br>*Field type*: Numeric field<br>*Variable name*: SDNN_m_1<br>*Field required*: Required<br>*Measurement Unit*: ms	ms
23.6	Total power, based on Lomb Scargle Periodogram, morning, night 1<br>*Field type*: Numeric field<br>*Variable name*: TP_LSP_m_1<br>*Field required*: Required<br>*Measurement Unit*: Hz	Hz
23.7	Low frequency power, based on Lomb Scargle Periodogram, morning, night 1<br>*Field type*: Numeric field<br>*Variable name*: LF_LSP_m_1<br>*Field required*: Required<br>*Measurement Unit*: Hz	Hz
23.8	High frequency power, based on Lomb Scargle Periodogram, morning, night 1<br>*Field type*: Numeric field<br>*Variable name*: HF_LSP_m_1<br>*Field required*: Required<br>*Measurement Unit*: Hz	Hz

23.9	LF/HF, based on Lomb Scargle Periodogram, morning, night 1<br>*Field type*: Numeric field<br>*Variable name*: LFHF_LSP_m_1<br>*Field required*: Required	
23.10	Total power, based on Fast Fourier Transformation, morning, night 1<br>*Field type*: Numeric field<br>*Variable name*: TP_FFT_m_1<br>*Field required*: Required<br>*Measurement Unit*: Hz	Hz
23.11	Low frequency power, based on Fast Fourier Transformation, morning, night 1<br>*Field type*: Numeric field<br>*Variable name*: LF_FFT_m_1<br>*Field required*: Required<br>*Measurement Unit*: Hz	Hz
23.12	High frequency power, based on Fast Fourier Transformation, morning, night 1<br>*Field type*: Numeric field<br>*Variable name*: HF_FFT_m_1<br>*Field required*: Required<br>*Measurement Unit*: Hz	Hz
23.13	LF/HF, based on Fast Fourier Transformation, morning, night 1<br>*Field type*: Numeric field<br>*Variable name*: LFHF_FFT_m_1<br>*Field required*: Required	

24. Baseline - Pre-sleep arousal scale

Number	Question	Answers
24.1	PSAS somatic night 1<br>*Field type*: Calculation<br>*Variable name*: PSAS_som_1<br>*Field required*: Not required<br>*Field min*: 8.00<br>*Field max*: 40.00	
24.2	PSAS cognitive night 1<br>*Field type*: Calculation<br>*Variable name*: PSAS_cog_1<br>*Field required*: Not required<br>*Field min*: 7.00<br>*Field max*: 35.00	

25. Baseline - Schlaffragebogen A baseline

Number	Question	Answers
25.1	Schlaffragebogen A 23d night 1<br>*Field type*: Calculation<br>*Variable name*: SFA_23d_1<br>*Field required*: Not required<br>*Field min*: 1.00<br>*Field max*: 5.00	
25.2	Schlaffragebogen A Allgemeine Schlafcharakterisierung night 1<br>*Field type*: Calculation<br>*Variable name*: SFA_ASC_1<br>*Field required*: Not required	
25.3	Schlaffragebogen A Vorzeitiges Aufwachen night 1<br>*Field type*: Calculation<br>*Variable name*: SFA_VZA_1<br>*Field required*: Not required	
25.4	Schlaffragebogen A Durchschlafschwierigkeiten night 1<br>*Field type*: Calculation<br>*Variable name*: SFA_DSS_1<br>*Field required*: Not required	
25.5	Schlaffragebogen A Einschlafschwierigkeiten night 1<br>*Field type*: Calculation<br>*Variable name*: SFA_ESS_1<br>*Field required*: Not required	
25.6	Schlaffragebogen A Schlafqualität night 1<br>*Field type*: Calculation<br>*Variable name*: SFA_SQ_1<br>*Field required*: Not required<br>*Field min*: 1.00<br>*Field max*: 5.00	
25.7	Schlaffragebogen A Gefühl des Erholtseins nach dem Schlaf night 1<br>*Field type*: Calculation<br>*Variable name*: SFA_GES_1<br>*Field required*: Not required<br>*Field min*: 1.00<br>*Field max*: 5.00	
25.8	Schlaffragebogen A Psychomotorische Symptome in der Schlafphase night 1<br>*Field type*: Calculation<br>*Variable name*: SFA_PSS_1<br>*Field required*: Not required<br>*Field min*: 1.00<br>*Field max*: 5.00	

25.9 Schlaffragebogen A Gesamtschlafdauer (Stunden) night 1
Field type: Calculation
Variable name: SFA_GSD_1
Field required: Not required
Field min: 1.00
Field max: 5.00

25.10 Schlaffragebogen A Psychische Ausgeglichenheit vor dem Schlafengehen night 1
Field type: Calculation
Variable name: SFA_PSYA_1
Field required: Not required
Field min: 1.00
Field max: 5.00

25.11 Schlaffragebogen A Psychisches Erschöpftsein vor dem Schlafengehen night 1
Field type: Calculation
Variable name: SFA_PSYE_1
Field required: Not required
Field min: 1.00
Field max: 5.00

26. Day 4 - Allocation

Number	Question	Answers
26.1	Allocation<br>*Field type*: Radiobutton<br>*Variable name*: Allocation<br>*Field required*: Required<br>*Option group name*: Allocation	◯ Control<br>◯ Aerobic exercise

27. Day 4 - Intervention

Number	Question	Answers
27.1	Befindlichkeitsskala activity pre-exercise<br>*Field type*: Calculation<br>*Variable name*: BFS_activity_1<br>*Field required*: Not required<br>*Field min*: 1.00<br>*Field max*: 5.00	
27.2	Befindlichkeitsskala elation pre-exercise<br>*Field type*: Calculation<br>*Variable name*: BFS_elation_1<br>*Field required*: Not required<br>*Field min*: 1.00<br>*Field max*: 5.00	
27.3	Befindlichkeitsskala contemplation pre-exercise<br>*Field type*: Calculation<br>*Variable name*: BFS_contemplation_1<br>*Field required*: Not required<br>*Field min*: 1.00<br>*Field max*: 5.00	
27.4	Befindlichkeitsskala calmness pre-exercise<br>*Field type*: Calculation<br>*Variable name*: BFS_calmness_1<br>*Field required*: Not required<br>*Field min*: 1.00<br>*Field max*: 5.00	
27.5	Befindlichkeitsskala fatigue pre-exercise<br>*Field type*: Calculation<br>*Variable name*: BFS_fatigue_1<br>*Field required*: Not required<br>*Field min*: 1.00<br>*Field max*: 5.00	
27.6	Befindlichkeitsskala depression pre-exercise<br>*Field type*: Calculation<br>*Variable name*: BFS_depression_1<br>*Field required*: Not required<br>*Field min*: 1.00<br>*Field max*: 5.00	
27.7	Befindlichkeitsskala anger pre-exercise<br>*Field type*: Calculation<br>*Variable name*: BFS_anger_1<br>*Field required*: Not required<br>*Field min*: 1.00<br>*Field max*: 5.00	
27.8	Befindlichkeitsskala excitement pre-exercise<br>*Field type*: Calculation<br>*Variable name*: BFS_excitement_1<br>*Field required*: Not required<br>*Field min*: 1.00<br>*Field max*: 5.00	

27.9 Befindlichkeitsskala activity post-exercise
Field type: Calculation
Variable name: BFS_activity_2
Field required: Not required
Field min: 1.00
Field max: 5.00

27.10 Befindlichkeitsskala elation post-exercise
Field type: Calculation
Variable name: BFS_elation_2
Field required: Not required
Field min: 1.00
Field max: 5.00

27.11 Befindlichkeitsskala contemplation post-exercise
Field type: Calculation
Variable name: BFS_contemplation_2
Field required: Not required
Field min: 1.00
Field max: 5.00

27.12 Befindlichkeitsskala calmness post-exercise
Field type: Calculation
Variable name: BFS_calmness_2
Field required: Not required
Field min: 1.00
Field max: 5.00

27.13 Befindlichkeitsskala fatigue post-exercise
Field type: Calculation
Variable name: BFS_fatigue_2
Field required: Not required
Field min: 1.00
Field max: 5.00

27.14 Befindlichkeitsskala depression post-exercise
Field type: Calculation
Variable name: BFS_depression_2
Field required: Not required
Field min: 1.00
Field max: 5.00

27.15 Befindlichkeitsskala anger post-exercise
Field type: Calculation
Variable name: BFS_anger_2
Field required: Not required
Field min: 1.00
Field max: 5.00

27.16 Befindlichkeitsskala excitement post-exercise
Field type: Calculation
Variable name: BFS_excitement_2
Field required: Not required
Field min: 1.00
Field max: 5.00

27.17	***If 'Allocation' is equal to 'Aerobic exercise' answer this question:***<br>Polar watch used for training<br>SM06: forschung@oberwaid.ch & Tatc93kW<br><br>SM07: gavin.brupbacher@oberwaid.ch & Loka75zY<br><br>V800 (Oberwaid) : sport@oberwaid.ch & OberwaidSport<br><br>*Field type*: Dropdown<br>*Variable name*: Polar_training<br>*Field required*: Required<br>*Field min*: 50.00<br>*Field max*: 220.00<br>*Measurement Unit*: beats per min<br>*Option group name*: Polar watch name	○ SM06<br>○ SM07<br>○ V800 Oberwaid
27.18	***If 'Allocation' is equal to 'Aerobic exercise' answer this question:***<br>Watt during intervention<br>*Field type*: Numeric field<br>*Variable name*: Watt_intervention<br>*Field required*: Required<br>*Field min*: 10.00<br>*Field max*: 400.00	
27.19	***If 'Allocation' is equal to 'Aerobic exercise' answer this question:***<br>Rate of perceived exertion after 5 min exercise at 80%IAS<br>*Field type*: Numeric field<br>*Variable name*: RPE_exercise_5<br>*Field required*: Required<br>*Field min*: 6.00<br>*Field max*: 20.00	
27.20	***If 'Allocation' is equal to 'Aerobic exercise' answer this question:***<br>Rate of perceived exertion after 15 min exercise at 80%IAS<br>*Field type*: Numeric field<br>*Variable name*: RPE_exercise_15<br>*Field required*: Required<br>*Field min*: 6.00<br>*Field max*: 20.00	
27.21	***If 'Allocation' is equal to 'Aerobic exercise' answer this question:***<br>Rate of perceived exertion after 30 min exercise at 80%IAS<br>*Field type*: Numeric field<br>*Variable name*: RPE_exercise_30<br>*Field required*: Required<br>*Field min*: 6.00<br>*Field max*: 20.00	

27.22	***If 'Allocation' is equal to 'Aerobic exercise' answer this question:***<br>Mean heart rate during exercise<br>*Field type*: Numeric field<br>*Variable name*: HR_exercise<br>*Field required*: Required<br>*Field min*: 50.00<br>*Field max*: 220.00<br>*Measurement Unit*: beats per min	beats per min
27.23	***If 'Allocation' is equal to 'Aerobic exercise' answer this question:***<br>Training_termination<br>*Field type*: Radiobutton<br>*Variable name*: Training_termination<br>*Field required*: Not required<br>*Option group name*: Yes/No	Yes<br>No
27.23.1	***If 'Training_termination' is equal to 'Yes' answer this question:***<br>Reason for training termination<br>*Field type*: Multiline Textfield<br>*Variable name*: Training_termination_reason<br>*Field required*: Required	

28. Follow-up - Polysomnography, sleep assessment 2

Number	Question	Answers
28.1	Date of followup polysomnography<br>*Field type*: Date<br>*Variable name*: Date_PSG_2<br>*Field required*: Required<br>*Measurement Unit*: min	(dd-mm-yyyy)
28.2	Sleep stage artefact duration<br>*Field type*: Numeric field<br>*Variable name*: Sleep_artefact_min_2<br>*Field required*: Required<br>*Measurement Unit*: min	min
28.3	Sleep stage artefact % total recording time<br>*Field type*: Numeric field<br>*Variable name*: Sleep_artefact_TRT_2<br>*Field required*: Required<br>*Measurement Unit*: %	%
28.4	Total recording time (TRT) night 2<br>*Field type*: Numeric field<br>*Variable name*: TRT_2<br>*Field required*: Required<br>*Measurement Unit*: min	min
28.5	Total sleep time (TST) night 2<br>*Field type*: Numeric field<br>*Variable name*: TST_2<br>*Field required*: Required<br>*Measurement Unit*: min	min
28.6	Sleep onset latency (SOL) night 2<br>*Field type*: Numeric field<br>*Variable name*: SOL_2<br>*Field required*: Required<br>*Measurement Unit*: min	min
28.7	Wake after sleep onset (WASO) night 2<br>*Field type*: Numeric field<br>*Variable name*: WASO_2<br>*Field required*: Required<br>*Measurement Unit*: min	min
28.8	Number of awakenings (NA) night 2<br>*Field type*: Numeric field<br>*Variable name*: NA_2<br>*Field required*: Required	
28.9	Sleep efficiency 1 (SE) night 2<br>*Field type*: Numeric field<br>*Variable name*: SE_2<br>*Field required*: Required<br>*Field min*: 0.00<br>*Field max*: 100.00<br>*Measurement Unit*: %	%

28.10 Wake min night 2 [ ] min
Field type: Numeric field
Variable name: Wake_min_2
Field required: Required
Measurement Unit: min

28.11 Wake %TRT night 2 [ ] %
Field type: Numeric field
Variable name: Wake_TRT_2
Field required: Required
Field min: 0.00
Field max: 100.00
Measurement Unit: %

28.12 N1 min night 2 [ ] min
Field type: Numeric field
Variable name: N1_min_2
Field required: Required
Measurement Unit: min

28.13 N1 %TRT night 2 [ ] %
Field type: Numeric field
Variable name: N1_TRT_2
Field required: Required
Field min: 0.00
Field max: 100.00
Measurement Unit: %

28.14 N1 %TST night 2
Field type: Calculation
Variable name: N1_TST_2
Field required: Not required
Field min: 0.00
Field max: 100.00
Measurement Unit: %

28.15 N2 min night 2 [ ] min
Field type: Numeric field
Variable name: N2_min_2
Field required: Required
Measurement Unit: min

28.16 N2 %TRT night 2 [ ] %
Field type: Numeric field
Variable name: N2_TRT_2
Field required: Required
Field min: 0.00
Field max: 100.00
Measurement Unit: %

28.17 N2 %TST night 2
Field type: Calculation
Variable name: N2_TST_2
Field required: Not required
Field min: 0.00
Field max: 100.00
Measurement Unit: %

28.18 N3 min night 2
Field type: Numeric field
Variable name: N3_min_2
Field required: Required
Measurement Unit: min

[ ] min

28.19 N3 %TRT night 2
Field type: Numeric field
Variable name: N3_TRT_2
Field required: Required
Field min: 0.00
Field max: 100.00
Measurement Unit: %

[ ] %

28.20 N3 %total sleep time night 2
Field type: Calculation
Variable name: N3_TST_2
Field required: Not required
Field min: 0.00
Field max: 100.00
Measurement Unit: %

28.21 Light Sleep min night 2
Field type: Calculation
Variable name: LS_min_2
Field required: Not required
Measurement Unit: min

28.22 Light Sleep %TST night 2
Field type: Calculation
Variable name: LS_TST_2
Field required: Not required
Field min: 0.00
Field max: 100.00
Measurement Unit: %

28.23 Slow wave sleep (SWS) min night 2
Field type: Numeric field
Variable name: SWS_min_2
Field required: Required
Measurement Unit: min

[ ] min

28.24 Slow wave sleep (SWS) %TRT night 2
Field type: Numeric field
Variable name: SWS_TRT_2
Field required: Required
Field min: 0.00
Field max: 100.00
Measurement Unit: %

[ ] %

28.25 Slow wave sleep (SWS) %TST night 2
Field type: Calculation
Variable name: SWS_TST_2
Field required: Not required
Field min: 0.00
Field max: 100.00
Measurement Unit: %

28.26	NREM (non-REM) sleep min night 2<br>*Field type*: Numeric field<br>*Variable name*: NREM_min_2<br>*Field required*: Required<br>*Measurement Unit*: min	min
28.27	NREM (non-REM) sleep %TRT night 2<br>*Field type*: Numeric field<br>*Variable name*: NREM_TRT_2<br>*Field required*: Required<br>*Field min*: 0.00<br>*Field max*: 100.00<br>*Measurement Unit*: %	%
28.28	NREM (non-REM) sleep %TST night 2<br>*Field type*: Calculation<br>*Variable name*: NREM_TST_2<br>*Field required*: Not required<br>*Field min*: 0.00<br>*Field max*: 100.00<br>*Measurement Unit*: %	
28.29	REM min night 2<br>*Field type*: Numeric field<br>*Variable name*: REM_min_2<br>*Field required*: Required<br>*Measurement Unit*: min	min
28.30	REM %TRT night 2<br>*Field type*: Numeric field<br>*Variable name*: REM_TRT_2<br>*Field required*: Required<br>*Field min*: 0.00<br>*Field max*: 100.00<br>*Measurement Unit*: %	%
28.31	REM % total sleep time night 2<br>*Field type*: Calculation<br>*Variable name*: REM_TST_2<br>*Field required*: Not required<br>*Field min*: 0.00<br>*Field max*: 100.00<br>*Measurement Unit*: %	
28.32	REMLAT min night 2<br>*Field type*: Numeric field<br>*Variable name*: REMLAT_min_2<br>*Field required*: Required<br>*Measurement Unit*: min	min
28.33	Stage shift index night 2<br>*Field type*: Numeric field<br>*Variable name*: SSI_2<br>*Field required*: Required	
28.34	Oxygen saturation artefact duration during sleep night 2<br>*Field type*: Numeric field<br>*Variable name*: SPO2_artefact_2<br>*Field required*: Required<br>*Measurement Unit*: min	min

28.35	Oxygen desaturation index from night 2<br>*Field type*: Numeric field<br>*Variable name*: ODI_2<br>*Field required*: Required	
28.36	Pulse transit time artefact duration during sleep night 2<br>*Field type*: Numeric field<br>*Variable name*: PTT_sleep_artefact_min_2<br>*Field required*: Required<br>*Measurement Unit*: min	min
28.37	Pulse transit time artefact duration during REM night 2<br>*Field type*: Numeric field<br>*Variable name*: PTT_REM_artefact_min_2<br>*Field required*: Required<br>*Measurement Unit*: min	min
28.38	Pulse transit time artefact duration during non-REM night 2<br>*Field type*: Numeric field<br>*Variable name*: PTT_NREM_artefact_min_2<br>*Field required*: Required<br>*Measurement Unit*: min	min
28.39	TST mean systolic BP night 2<br>*Field type*: Numeric field<br>*Variable name*: TST_Sys_BP_2<br>*Field required*: Required<br>*Measurement Unit*: mmHg	mmHg
28.40	TST mean diastolic BP night 2<br>*Field type*: Numeric field<br>*Variable name*: TST_Dia_BP_2<br>*Field required*: Required<br>*Measurement Unit*: mmHg	mmHg
28.41	NREM mean systolic BP night 2<br>*Field type*: Numeric field<br>*Variable name*: NREM_Sys_BP_2<br>*Field required*: Required<br>*Measurement Unit*: mmHg	mmHg
28.42	NREM mean diastolic BP night 2<br>*Field type*: Numeric field<br>*Variable name*: NREM_Dia_BP_2<br>*Field required*: Required<br>*Measurement Unit*: mmHg	mmHg
28.43	REM mean systolic BP night 2<br>*Field type*: Numeric field<br>*Variable name*: REM_Sys_BP_2<br>*Field required*: Required<br>*Measurement Unit*: mmHg	mmHg
28.44	REM mean diastolic BP night 2<br>*Field type*: Numeric field<br>*Variable name*: REM_Dia_BP_2<br>*Field required*: Required<br>*Measurement Unit*: mmHg	mmHg

28.45 Mean arterial pressure night 2
Field type: Calculation
Variable name: MAP_night_2
Field required: Not required

29. Follow-up - Pre sleep HRV, sleep assessment 2

Number	Question	Answers
29.1	Start and stop time of pre-sleep segment, night 2<br>*Field type*: Textfield<br>*Variable name*: Time_PS_2<br>*Field required*: Required<br>*Measurement Unit*: hh:mm:ss-hh:mm:ss	[ ] hh:mm:ss-hh:mm:ss
29.2	Artefact of pre-sleep segment, night 2<br>*Field type*: Numeric field<br>*Variable name*: Artefact_PS_2<br>*Field required*: Required<br>*Field min*: 0.00<br>*Field max*: 100.00<br>*Measurement Unit*: %	[ ] %
29.3	Mean heart rate, Pre-sleep, night 2<br>*Field type*: Numeric field<br>*Variable name*: meanHR_PS_2<br>*Field required*: Required<br>*Measurement Unit*: beats per min	[ ] beats per min
29.4	RMSSD Pre-sleep HRV night 2<br>*Field type*: Numeric field<br>*Variable name*: RMSSD_PS_2<br>*Field required*: Required<br>*Measurement Unit*: ms	[ ] ms
29.5	SDNN Pre-sleep HRV night 2<br>*Field type*: Numeric field<br>*Variable name*: SDNN_PS_2<br>*Field required*: Required<br>*Measurement Unit*: ms	[ ] ms
29.6	Total power, based on Lomb Scargle Periodogram, Pre-sleep, night 2<br>*Field type*: Numeric field<br>*Variable name*: TP_LSP_PS_2<br>*Field required*: Required<br>*Measurement Unit*: Hz	[ ] Hz
29.7	Low frequency power, based on Lomb Scargle Periodogram, Pre-sleep, night 2<br>*Field type*: Numeric field<br>*Variable name*: LF_LSP_PS_2<br>*Field required*: Required<br>*Measurement Unit*: Hz	[ ] Hz
29.8	High frequency power, based on Lomb Scargle Periodogram, Pre-sleep, night 2<br>*Field type*: Numeric field<br>*Variable name*: HF_LSP_PS_2<br>*Field required*: Required<br>*Measurement Unit*: Hz	[ ] Hz

29.9	LF/HF, based on Lomb Scargle Periodogram, Pre-sleep, night 2<br>*Field type*: Numeric field<br>*Variable name*: LFHF_LSP_PS_2<br>*Field required*: Required	
29.10	Total power, based on Fast Fourier Transformation, Pre-sleep night 2<br>*Field type*: Numeric field<br>*Variable name*: TP_FFT_PS_2<br>*Field required*: Required<br>*Measurement Unit*: Hz	Hz
29.11	Low frequency power, based on Fast Fourier Transformation, Pre-sleep night 2<br>*Field type*: Numeric field<br>*Variable name*: LF_FFT_PS_2<br>*Field required*: Required<br>*Measurement Unit*: Hz	Hz
29.12	High frequency power, based on Fast Fourier Transformation, Pre-sleep night 2<br>*Field type*: Numeric field<br>*Variable name*: HF_FFT_PS_2<br>*Field required*: Required<br>*Measurement Unit*: Hz	Hz
29.13	LF/HF, based on Fast Fourier Transformation, Pre-sleep night 2<br>*Field type*: Numeric field<br>*Variable name*: LFHF_FFT_PS_2<br>*Field required*: Required	

30. Follow-up - Nocturnal hour 1 HRV, sleep assessment 2

Number	Question	Answers
30.1	Start and stop time of nocturnal hour 1 segment, night 2<br>*Field type*: Textfield<br>*Variable name*: Time_h1_2<br>*Field required*: Required<br>*Measurement Unit*: hh:mm:ss-hh:mm:ss	[ ] hh:mm:ss-hh:mm:ss
30.2	Artefact of nocturnal hour 1 segment, night 2<br>*Field type*: Numeric field<br>*Variable name*: Artefact_h1_2<br>*Field required*: Required<br>*Field min*: 0.00<br>*Field max*: 100.00<br>*Measurement Unit*: %	[ ] %
30.3	Mean heart rate, nocturnal hour 1, night 2<br>*Field type*: Numeric field<br>*Variable name*: meanHR_h1_2<br>*Field required*: Required<br>*Measurement Unit*: beats per min	[ ] beats per min
30.4	RMSSD, Nocturnal hour 1, night 2<br>*Field type*: Numeric field<br>*Variable name*: RMSSD_h1_2<br>*Field required*: Required<br>*Measurement Unit*: ms	[ ] ms
30.5	SDNN, Nocturnal hour 1, night 2<br>*Field type*: Numeric field<br>*Variable name*: SDNN_h1_2<br>*Field required*: Required<br>*Measurement Unit*: ms	[ ] ms
30.6	SDANN, Nocturnal hour 1, night 2<br>*Field type*: Numeric field<br>*Variable name*: SDANN_h1_2<br>*Field required*: Required<br>*Measurement Unit*: ms	[ ] ms
30.7	Total power, based on Lomb Scargle Periodogram, Nocturnal hour 1, night 2<br>*Field type*: Numeric field<br>*Variable name*: TP_LSP_h1_2<br>*Field required*: Required<br>*Measurement Unit*: Hz	[ ] Hz
30.8	Low frequency power, based on Lomb Scargle Periodogram, Nocturnal hour 1, night 2<br>*Field type*: Numeric field<br>*Variable name*: LF_LSP_h1_2<br>*Field required*: Required<br>*Measurement Unit*: Hz	[ ] Hz

30.9	High frequency power, based on Lomb Scargle Periodogram, Nocturnal hour 1, night 2<br>*Field type*: Numeric field<br>*Variable name*: HF_LSP_h1_2<br>*Field required*: Required<br>*Measurement Unit*: Hz	Hz
30.10	LF/HF, based on Lomb Scargle Periodogram, Nocturnal hour 1, night 2<br>*Field type*: Numeric field<br>*Variable name*: LFHF_LSP_h1_2<br>*Field required*: Required	
30.11	Total power, based on Fast Fourier Transformation, Nocturnal hour 1, night 2<br>*Field type*: Numeric field<br>*Variable name*: TP_FFT_h1_2<br>*Field required*: Required<br>*Measurement Unit*: Hz	Hz
30.12	Low frequency power, based on Fast Fourier Transformation, Nocturnal hour 1, night 1<br>*Field type*: Numeric field<br>*Variable name*: LF_FFT_h1_2<br>*Field required*: Required<br>*Measurement Unit*: Hz	Hz
30.13	High frequency power, based on Fast Fourier Transformation, Nocturnal hour 1, night 2<br>*Field type*: Numeric field<br>*Variable name*: HF_FFT_h1_2<br>*Field required*: Required<br>*Measurement Unit*: Hz	Hz
30.14	LF/HF, based on Fast Fourier Transformation, Nocturnal hour 1, night 2<br>*Field type*: Numeric field<br>*Variable name*: LFHF_FFT_h1_2<br>*Field required*: Required	

31. Follow-up - Nocturnal hour 2 HRV, sleep assessment 2

Number	Question	Answers
31.1	Start and stop time of nocturnal hour 2 segment, night 2<br>*Field type*: Textfield<br>*Variable name*: Time_h2_2<br>*Field required*: Required<br>*Measurement Unit*: hh:mm:ss-hh:mm:ss	[ ] hh:mm:ss-hh:mm:ss
31.2	Artefact of nocturnal hour 2 segment, night 2<br>*Field type*: Numeric field<br>*Variable name*: Artefact_h2_2<br>*Field required*: Required<br>*Field min*: 0.00<br>*Field max*: 100.00<br>*Measurement Unit*: %	[ ] %
31.3	Mean heart rate, nocturnal hour 2, night 2<br>*Field type*: Numeric field<br>*Variable name*: meanHR_h2_2<br>*Field required*: Required<br>*Measurement Unit*: beats per min	[ ] beats per min
31.4	RMSSD, Nocturnal hour 2, night 2<br>*Field type*: Numeric field<br>*Variable name*: RMSSD_h2_2<br>*Field required*: Required<br>*Measurement Unit*: ms	[ ] ms
31.5	SDNN, Nocturnal hour 2, night 2<br>*Field type*: Numeric field<br>*Variable name*: SDNN_h2_2<br>*Field required*: Required<br>*Measurement Unit*: ms	[ ] ms
31.6	SDANN, Nocturnal hour 2, night 2<br>*Field type*: Numeric field<br>*Variable name*: SDANN_h2_2<br>*Field required*: Required<br>*Measurement Unit*: ms	[ ] ms
31.7	Total power, based on Lomb Scargle Periodogram, Nocturnal hour 2, night 2<br>*Field type*: Numeric field<br>*Variable name*: TP_LSP_h2_2<br>*Field required*: Required<br>*Measurement Unit*: Hz	[ ] Hz
31.8	Low frequency power, based on Lomb Scargle Periodogram, Nocturnal hour 2, night 2<br>*Field type*: Numeric field<br>*Variable name*: LF_LSP_h2_2<br>*Field required*: Required<br>*Measurement Unit*: Hz	[ ] Hz

31.9	High frequency power, based on Lomb Scargle Periodogram, Nocturnal hour 2, night 2<br>*Field type*: Numeric field<br>*Variable name*: HF_LSP_h2_2<br>*Field required*: Required<br>*Measurement Unit*: Hz	[ ] Hz
31.10	LF/HF, based on Lomb Scargle Periodogram, Nocturnal hour 2, night 2<br>*Field type*: Numeric field<br>*Variable name*: LFHF_LSP_h2_2<br>*Field required*: Required	[ ]
31.11	Total power, based on Fast Fourier Transformation, Nocturnal hour 2, night 2<br>*Field type*: Numeric field<br>*Variable name*: TP_FFT_h2_2<br>*Field required*: Required<br>*Measurement Unit*: Hz	[ ] Hz
31.12	Low frequency power, based on Fast Fourier Transformation, Nocturnal hour 2, night 2<br>*Field type*: Numeric field<br>*Variable name*: LF_FFT_h2_2<br>*Field required*: Required<br>*Measurement Unit*: Hz	[ ] Hz
31.13	High frequency power, based on Fast Fourier Transformation, Nocturnal hour 2, night 2<br>*Field type*: Numeric field<br>*Variable name*: HF_FFT_h2_2<br>*Field required*: Required<br>*Measurement Unit*: Hz	[ ] Hz
31.14	LF/HF, based on Fast Fourier Transformation, Nocturnal hour 2, night 2<br>*Field type*: Numeric field<br>*Variable name*: LFHF_FFT_h2_2<br>*Field required*: Required	[ ]

32. Follow-up - Nocturnal hour 3 HRV, sleep assessment 2

Number	Question	Answers
32.1	Start and stop time of nocturnal hour 3 segment, night 2<br>*Field type*: Textfield<br>*Variable name*: Time_h3_2<br>*Field required*: Required<br>*Measurement Unit*: hh:mm:ss-hh:mm:ss	[ ] hh:mm:ss-hh:mm:ss
32.2	Artefact of nocturnal hour 3 segment, night 2<br>*Field type*: Numeric field<br>*Variable name*: Artefact_h3_2<br>*Field required*: Required<br>*Field min*: 0.00<br>*Field max*: 100.00<br>*Measurement Unit*: %	[ ] %
32.3	Mean heart rate, nocturnal hour 3, night 2<br>*Field type*: Numeric field<br>*Variable name*: meanHR_h3_2<br>*Field required*: Required<br>*Measurement Unit*: beats per min	[ ] beats per min
32.4	RMSSD, Nocturnal hour 3, night 2<br>*Field type*: Numeric field<br>*Variable name*: RMSSD_h3_2<br>*Field required*: Required<br>*Measurement Unit*: ms	[ ] ms
32.5	SDNN, Nocturnal hour 3, night 2<br>*Field type*: Numeric field<br>*Variable name*: SDNN_h3_2<br>*Field required*: Required<br>*Measurement Unit*: ms	[ ] ms
32.6	SDANN, Nocturnal hour 3, night 2<br>*Field type*: Numeric field<br>*Variable name*: SDANN_h3_2<br>*Field required*: Required<br>*Measurement Unit*: ms	[ ] ms
32.7	Total power, based on Lomb Scargle Periodogram, Nocturnal hour 3, night 2<br>*Field type*: Numeric field<br>*Variable name*: TP_LSP_h3_2<br>*Field required*: Required<br>*Measurement Unit*: Hz	[ ] Hz
32.8	Low frequency power, based on Lomb Scargle Periodogram, Nocturnal hour 3, night 2<br>*Field type*: Numeric field<br>*Variable name*: LF_LSP_h3_2<br>*Field required*: Required<br>*Measurement Unit*: Hz	[ ] Hz

32.9	High frequency power, based on Lomb Scargle Periodogram, Nocturnal hour 3, night 2<br>*Field type*: Numeric field<br>*Variable name*: HF_LSP_h3_2<br>*Field required*: Required<br>*Measurement Unit*: Hz		Hz
32.10	LF/HF, based on Lomb Scargle Periodogram, Nocturnal hour 3, night 2<br>*Field type*: Numeric field<br>*Variable name*: LFHF_LSP_h3_2<br>*Field required*: Required		
32.11	Total power, based on Fast Fourier Transformation, Nocturnal hour 3, night 2<br>*Field type*: Numeric field<br>*Variable name*: TP_FFT_h3_2<br>*Field required*: Required<br>*Measurement Unit*: Hz		Hz
32.12	Low frequency power, based on Fast Fourier Transformation, Nocturnal hour 3, night 2<br>*Field type*: Numeric field<br>*Variable name*: LF_FFT_h3_2<br>*Field required*: Required<br>*Measurement Unit*: Hz		Hz
32.13	High frequency power, based on Fast Fourier Transformation, Nocturnal hour 3, night 2<br>*Field type*: Numeric field<br>*Variable name*: HF_FFT_h3_2<br>*Field required*: Required<br>*Measurement Unit*: Hz		Hz
32.14	LF/HF, based on Fast Fourier Transformation, Nocturnal hour 3, night 2<br>*Field type*: Numeric field<br>*Variable name*: LFHF_FFT_h3_2<br>*Field required*: Required		

33. Follow-up - Nocturnal hour 4 HRV, sleep assessment 2

Number	Question	Answers
33.1	Start and stop time of nocturnal hour 4 segment, night 2<br>*Field type*: Textfield<br>*Variable name*: Time_h4_2<br>*Field required*: Required<br>*Measurement Unit*: hh:mm:ss-hh:mm:ss	[ ] hh:mm:ss-hh:mm:ss
33.2	Artefact of nocturnal hour 4 segment, night 2<br>*Field type*: Numeric field<br>*Variable name*: Artefact_h4_2<br>*Field required*: Required<br>*Field min*: 0.00<br>*Field max*: 100.00<br>*Measurement Unit*: %	[ ] %
33.3	Mean heart rate, nocturnal hour 4, night 2<br>*Field type*: Numeric field<br>*Variable name*: meanHR_h4_2<br>*Field required*: Required<br>*Measurement Unit*: beats per min	[ ] beats per min
33.4	RMSSD, Nocturnal hour 4, night 2<br>*Field type*: Numeric field<br>*Variable name*: RMSSD_h4_2<br>*Field required*: Required<br>*Measurement Unit*: ms	[ ] ms
33.5	SDNN, Nocturnal hour 4, night 2<br>*Field type*: Numeric field<br>*Variable name*: SDNN_h4_2<br>*Field required*: Required<br>*Measurement Unit*: ms	[ ] ms
33.6	SDANN, Nocturnal hour 4, night 2<br>*Field type*: Numeric field<br>*Variable name*: SDANN_h4_2<br>*Field required*: Required<br>*Measurement Unit*: ms	[ ] ms
33.7	Total power, based on Lomb Scargle Periodogram, Nocturnal hour 4, night 2<br>*Field type*: Numeric field<br>*Variable name*: TP_LSP_h4_2<br>*Field required*: Required<br>*Measurement Unit*: Hz	[ ] Hz
33.8	Low frequency power, based on Lomb Scargle Periodogram, Nocturnal hour 4, night 2<br>*Field type*: Numeric field<br>*Variable name*: LF_LSP_h4_2<br>*Field required*: Required<br>*Measurement Unit*: Hz	[ ] Hz

33.9	High frequency power, based on Lomb Scargle Periodogram, Nocturnal hour 4, night 2<br>*Field type*: Numeric field<br>*Variable name*: HF_LSP_h4_2<br>*Field required*: Required<br>*Measurement Unit*: Hz	Hz
33.10	LF/HF, based on Lomb Scargle Periodogram, Nocturnal hour 4, night 2<br>*Field type*: Numeric field<br>*Variable name*: LFHF_LSP_h4_2<br>*Field required*: Required	
33.11	Total power, based on Fast Fourier Transformation, Nocturnal hour 4, night 2<br>*Field type*: Numeric field<br>*Variable name*: TP_FFT_h4_2<br>*Field required*: Required<br>*Measurement Unit*: Hz	Hz
33.12	Low frequency power, based on Fast Fourier Transformation, Nocturnal hour 4, night 2<br>*Field type*: Numeric field<br>*Variable name*: LF_FFT_h4_2<br>*Field required*: Required<br>*Measurement Unit*: Hz	Hz
33.13	High frequency power, based on Fast Fourier Transformation, Nocturnal hour 4, night 2<br>*Field type*: Numeric field<br>*Variable name*: HF_FFT_h4_2<br>*Field required*: Required<br>*Measurement Unit*: Hz	Hz
33.14	LF/HF, based on Fast Fourier Transformation, Nocturnal hour 4, night 2<br>*Field type*: Numeric field<br>*Variable name*: LFHF_FFT_h4_2<br>*Field required*: Required	

34. Follow-up - Nocturnal hour 5 HRV, sleep assessment 2

Number	Question	Answers
34.1	Start and stop time of nocturnal hour 5 segment, night 2<br>*Field type*: Textfield<br>*Variable name*: Time_h5_2<br>*Field required*: Required<br>*Measurement Unit*: hh:mm:ss-hh:mm:ss	[ ] hh:mm:ss-hh:mm:ss
34.2	Artefact of nocturnal hour 5 segment, night 2<br>*Field type*: Numeric field<br>*Variable name*: Artefact_h5_2<br>*Field required*: Required<br>*Field min*: 0.00<br>*Field max*: 100.00<br>*Measurement Unit*: %	[ ] %
34.3	Mean heart rate, nocturnal hour 5, night 2<br>*Field type*: Numeric field<br>*Variable name*: meanHR_h5_2<br>*Field required*: Required<br>*Measurement Unit*: beats per min	[ ] beats per min
34.4	RMSSD, Nocturnal hour 5, night 2<br>*Field type*: Numeric field<br>*Variable name*: RMSSD_h5_2<br>*Field required*: Required<br>*Measurement Unit*: ms	[ ] ms
34.5	SDNN, Nocturnal hour 5, night 2<br>*Field type*: Numeric field<br>*Variable name*: SDNN_h5_2<br>*Field required*: Required<br>*Measurement Unit*: ms	[ ] ms
34.6	SDANN, Nocturnal hour 5, night 2<br>*Field type*: Numeric field<br>*Variable name*: SDANN_h5_2<br>*Field required*: Required<br>*Measurement Unit*: ms	[ ] ms
34.7	Total power, based on Lomb Scargle Periodogram, Nocturnal hour 5, night 2<br>*Field type*: Numeric field<br>*Variable name*: TP_LSP_h5_2<br>*Field required*: Required<br>*Measurement Unit*: Hz	[ ] Hz
34.8	Low frequency power, based on Lomb Scargle Periodogram, Nocturnal hour 5, night 2<br>*Field type*: Numeric field<br>*Variable name*: LF_LSP_h5_2<br>*Field required*: Required<br>*Measurement Unit*: Hz	[ ] Hz

34.9	High frequency power, based on Lomb Scargle Periodogram, Nocturnal hour 5, night 2<br>*Field type*: Numeric field<br>*Variable name*: HF_LSP_h5_2<br>*Field required*: Required<br>*Measurement Unit*: Hz	[ ] Hz
34.10	LF/HF, based on Lomb Scargle Periodogram, Nocturnal hour 5, night 2<br>*Field type*: Numeric field<br>*Variable name*: LFHF_LSP_h5_2<br>*Field required*: Required	[ ]
34.11	Total power, based on Fast Fourier Transformation, Nocturnal hour 5, night 2<br>*Field type*: Numeric field<br>*Variable name*: TP_FFT_h5_2<br>*Field required*: Required<br>*Measurement Unit*: Hz	[ ] Hz
34.12	Low frequency power, based on Fast Fourier Transformation, Nocturnal hour 5, night 2<br>*Field type*: Numeric field<br>*Variable name*: LF_FFT_h5_2<br>*Field required*: Required<br>*Measurement Unit*: Hz	[ ] Hz
34.13	High frequency power, based on Fast Fourier Transformation, Nocturnal hour 5, night 2<br>*Field type*: Numeric field<br>*Variable name*: HF_FFT_h5_2<br>*Field required*: Required<br>*Measurement Unit*: Hz	[ ] Hz
34.14	LF/HF, based on Fast Fourier Transformation, Nocturnal hour 5, night 2<br>*Field type*: Numeric field<br>*Variable name*: LFHF_FFT_h5_2<br>*Field required*: Required	[ ]

35. Follow-up - Nocturnal hour 6 HRV, sleep assessment 2

Number	Question	Answers
35.1	Start and stop time of nocturnal hour 6 segment, night 2<br>*Field type*: Textfield<br>*Variable name*: Time_h6_2<br>*Field required*: Required<br>*Measurement Unit*: hh:mm:ss-hh:mm:ss	[ ] hh:mm:ss-hh:mm:ss
35.2	Artefact of nocturnal hour 6 segment, night 2<br>*Field type*: Numeric field<br>*Variable name*: Artefact_h6_2<br>*Field required*: Required<br>*Field min*: 0.00<br>*Field max*: 100.00<br>*Measurement Unit*: %	[ ] %
35.3	Mean heart rate, nocturnal hour 6, night 2<br>*Field type*: Numeric field<br>*Variable name*: meanHR_h6_2<br>*Field required*: Required<br>*Measurement Unit*: beats per min	[ ] beats per min
35.4	RMSSD, Nocturnal hour 6, night 2<br>*Field type*: Numeric field<br>*Variable name*: RMSSD_h6_2<br>*Field required*: Required<br>*Measurement Unit*: ms	[ ] ms
35.5	SDNN, Nocturnal hour 6, night 2<br>*Field type*: Numeric field<br>*Variable name*: SDNN_h6_2<br>*Field required*: Required<br>*Measurement Unit*: ms	[ ] ms
35.6	SDANN, Nocturnal hour 6, night 2<br>*Field type*: Numeric field<br>*Variable name*: SDANN_h6_2<br>*Field required*: Required<br>*Measurement Unit*: ms	[ ] ms
35.7	Total power, based on Lomb Scargle Periodogram, Nocturnal hour 6, night 2<br>*Field type*: Numeric field<br>*Variable name*: TP_LSP_h6_2<br>*Field required*: Required<br>*Measurement Unit*: Hz	[ ] Hz
35.8	Low frequency power, based on Lomb Scargle Periodogram, Nocturnal hour 6, night 2<br>*Field type*: Numeric field<br>*Variable name*: LF_LSP_h6_2<br>*Field required*: Required<br>*Measurement Unit*: Hz	[ ] Hz

35.9	High frequency power, based on Lomb Scargle Periodogram, Nocturnal hour 6, night 2<br>*Field type*: Numeric field<br>*Variable name*: HF_LSP_h6_2<br>*Field required*: Required<br>*Measurement Unit*: Hz	Hz
35.10	LF/HF, based on Lomb Scargle Periodogram, Nocturnal hour 6, night 2<br>*Field type*: Numeric field<br>*Variable name*: LFHF_LSP_h6_2<br>*Field required*: Required	
35.11	Total power, based on Fast Fourier Transformation, Nocturnal hour 6, night 2<br>*Field type*: Numeric field<br>*Variable name*: TP_FFT_h6_2<br>*Field required*: Required<br>*Measurement Unit*: Hz	Hz
35.12	Low frequency power, based on Fast Fourier Transformation, Nocturnal hour 6, night 2<br>*Field type*: Numeric field<br>*Variable name*: LF_FFT_h6_2<br>*Field required*: Required<br>*Measurement Unit*: Hz	Hz
35.13	High frequency power, based on Fast Fourier Transformation, Nocturnal hour 6, night 2<br>*Field type*: Numeric field<br>*Variable name*: HF_FFT_h6_2<br>*Field required*: Required<br>*Measurement Unit*: Hz	Hz
35.14	LF/HF, based on Fast Fourier Transformation, Nocturnal hour 6, night 2<br>*Field type*: Numeric field<br>*Variable name*: LFHF_FFT_h6_2<br>*Field required*: Required	

36. Follow-up - Morning HRV, sleep assessment 2

Number	Question	Answers
36.1	Start and stop time of morning segment, night 2<br>*Field type*: Textfield<br>*Variable name*: Time_m_2<br>*Field required*: Required<br>*Measurement Unit*: hh:mm:ss-hh:mm:ss	[ ] hh:mm:ss-hh:mm:ss
36.2	Artefact of morning segment, night 2<br>*Field type*: Numeric field<br>*Variable name*: Artefact_m_2<br>*Field required*: Required<br>*Field min*: 0.00<br>*Field max*: 100.00<br>*Measurement Unit*: %	[ ] %
36.3	Mean heart rate, morning, night 2<br>*Field type*: Numeric field<br>*Variable name*: meanHR_m_2<br>*Field required*: Required<br>*Measurement Unit*: beats per min	[ ] beats per min
36.4	RMSSD morning HRV night 2<br>*Field type*: Numeric field<br>*Variable name*: RMSSD_m_2<br>*Field required*: Required<br>*Measurement Unit*: ms	[ ] ms
36.5	SDNN morning HRV night 2<br>*Field type*: Numeric field<br>*Variable name*: SDNN_m_2<br>*Field required*: Required<br>*Measurement Unit*: ms	[ ] ms
36.6	Total power, based on Lomb Scargle Periodogram, morning, night 2<br>*Field type*: Numeric field<br>*Variable name*: TP_LSP_m_2<br>*Field required*: Required<br>*Measurement Unit*: Hz	[ ] Hz
36.7	Low frequency power, based on Lomb Scargle Periodogram, morning, night 2<br>*Field type*: Numeric field<br>*Variable name*: LF_LSP_m_2<br>*Field required*: Required<br>*Measurement Unit*: Hz	[ ] Hz
36.8	High frequency power, based on Lomb Scargle Periodogram, morning, night 2<br>*Field type*: Numeric field<br>*Variable name*: HF_LSP_m_2<br>*Field required*: Required<br>*Measurement Unit*: Hz	[ ] Hz

36.9	LF/HF, based on Lomb Scargle Periodogram, morning, night 2<br>*Field type*: Numeric field<br>*Variable name*: LFHF_LSP_m_2<br>*Field required*: Required		
36.10	Total power, based on Fast Fourier Transformation, morning, night 2<br>*Field type*: Numeric field<br>*Variable name*: TP_FFT_m_2<br>*Field required*: Required<br>*Measurement Unit*: Hz		Hz
36.11	Low frequency power, based on Fast Fourier Transformation, morning, night 2<br>*Field type*: Numeric field<br>*Variable name*: LF_FFT_m_2<br>*Field required*: Required<br>*Measurement Unit*: Hz		Hz
36.12	High frequency power, based on Fast Fourier Transformation, morning, night 2<br>*Field type*: Numeric field<br>*Variable name*: HF_FFT_m_2<br>*Field required*: Required<br>*Measurement Unit*: Hz		Hz
36.13	LF/HF, based on Fast Fourier Transformation, morning, night 2<br>*Field type*: Numeric field<br>*Variable name*: LFHF_FFT_m_2<br>*Field required*: Required		

37. Follow-up - Pre-sleep arousal scale

Number	Question	Answers
37.1	PSAS somatic night 2<br>*Field type*: Calculation<br>*Variable name*: PSAS_som_2<br>*Field required*: Not required<br>*Field min*: 8.00<br>*Field max*: 40.00	
37.2	PSAS cognitive night 2<br>*Field type*: Calculation<br>*Variable name*: PSAS_cog_2<br>*Field required*: Not required<br>*Field min*: 7.00<br>*Field max*: 35.00	

38. Follow-up - Schlaffragebogen A follow-up

Number	Question	Answers
38.1	Schlaffragebogen A 23d night 2<br>*Field type*: Calculation<br>*Variable name*: SFA_23d_2<br>*Field required*: Not required<br>*Field min*: 1.00<br>*Field max*: 5.00	
38.2	Schlaffragebogen A Allgemeine Schlafcharakterisierung night 2<br>*Field type*: Calculation<br>*Variable name*: SFA_ASC_2<br>*Field required*: Not required	
38.3	Schlaffragebogen A Vorzeitiges Aufwachen night 2<br>*Field type*: Calculation<br>*Variable name*: SFA_VZA_2<br>*Field required*: Not required	
38.4	Schlaffragebogen A Durchschlafschwierigkeiten night 2<br>*Field type*: Calculation<br>*Variable name*: SFA_DSS_2<br>*Field required*: Not required	
38.5	Schlaffragebogen A Einschlafschwierigkeiten night 2<br>*Field type*: Calculation<br>*Variable name*: SFA_ESS_2<br>*Field required*: Not required	
38.6	Schlaffragebogen A Schlafqualität night 2<br>*Field type*: Calculation<br>*Variable name*: SFA_SQ_2<br>*Field required*: Not required<br>*Field min*: 1.00<br>*Field max*: 5.00	
38.7	Schlaffragebogen A Gefühl des Erholtseins nach dem Schlaf night 2<br>*Field type*: Calculation<br>*Variable name*: SFA_GES_2<br>*Field required*: Not required<br>*Field min*: 1.00<br>*Field max*: 5.00	
38.8	Schlaffragebogen A Psychomotorische Symptome in der Schlafphase night 2<br>*Field type*: Calculation<br>*Variable name*: SFA_PSS_2<br>*Field required*: Not required<br>*Field min*: 1.00<br>*Field max*: 5.00	

38.9	Schlaffragebogen A Gesamtschlafdauer (Stunden) night 2<br>*Field type*: Calculation<br>*Variable name*: SFA_GSD_2<br>*Field required*: Not required<br>*Field min*: 1.00<br>*Field max*: 5.00
38.10	Schlaffragebogen A Psychische Ausgeglichenheit vor dem Schlafengehen night 2<br>*Field type*: Calculation<br>*Variable name*: SFA_PSYA_2<br>*Field required*: Not required<br>*Field min*: 1.00<br>*Field max*: 5.00
38.11	Schlaffragebogen A Psychisches Erschöpftsein vor dem Schlafengehen night 2<br>*Field type*: Calculation<br>*Variable name*: SFA_PSYE_2<br>*Field required*: Not required<br>*Field min*: 1.00<br>*Field max*: 5.00

39. Day 5 - Daytime sleepiness 0800 hrs

Number	Question	Answers
39.1	Daytime sleepiness 0800 hrs<br>*Field type*: Calculation<br>*Variable name*: SSS_0800hrs<br>*Field required*: Not required	

40. Day 5 - Daytime sleepiness 1200 hrs

Number	Question	Answers
40.1	Daytime sleepiness 1200 hrs<br>*Field type*: Calculation<br>*Variable name*: SSS_1200hrs<br>*Field required*: Not required	

41. Day 5 - Daytime sleepiness 1600 hrs

Number	Question	Answers
41.1	Daytime sleepiness 1600 hrs<br>*Field type*: Calculation<br>*Variable name*: SSS_1600hrs<br>*Field required*: Not required	

42. Day 5 - Daytime sleepiness 2000 hrs

Number	Question	Answers
42.1	Daytime sleepiness 2000 hrs<br>*Field type*: Radiobutton<br>*Variable name*: SSS_2000hrs<br>*Field required*: Not required<br>*Option group name*: SSS	◯ Fühle mich aktiv und vital; vollkommen wach<br>◯ Bin voll da, jedoch nicht auf den Höhepunkt; kann mich konzentrieren<br>◯ Entspannt; wach; nicht voll aufmerksam, ansprechbar<br>◯ Etwas dösig; nicht auf dem Höhepunkt; etwas schlapp<br>◯ Dösig; verliere das Interesse, wach zu bleiben; verlangsamt<br>◯ Schläfrig; möchte mich hinlegen; kämpfe gegen den Schlaf; benebelt<br>◯ Fast träumend; schlafe bald ein; kein Bemühen mehr, wach zu bleibe

43. Days 3-5 - Adherence

Number	Question	Answers
43.1	Name of vivofit wristband<br>SM02: mobigame2016@gmail.com & Mobigame_SM02<br><br>SM03: mobigamesm03@gmail.com & Mobigame_SM03<br><br>SM01 (nur in Reserve): mobigamesm01@gmail.com & Mobigame_SM01<br><br>*Field type*: Dropdown<br>*Variable name*: Vivofit_name<br>*Field required*: Required<br>*Option group name*: Vivofit name	○ SM01<br>○ SM02<br>○ SM03
43.2	Steps on day 3<br>*Field type*: Numeric field<br>*Variable name*: Steps_day3<br>*Field required*: Required	
43.3	Steps on day 4<br>*Field type*: Numeric field<br>*Variable name*: Steps_day4<br>*Field required*: Required	
43.4	Steps on day 5<br>*Field type*: Numeric field<br>*Variable name*: Steps_day5<br>*Field required*: Required	

44. Any point during trial - Retention

Number	Question	Answers
44.1	Dropout of patient<br>*Field type*: Radiobutton<br>*Variable name*: Patient_dropout<br>*Field required*: Not required<br>*Option group name*: Yes/No	○ Yes<br>○ No
44.1.1	***If 'Dropout of patient' is equal to 'Yes' answer this question:***<br>Patient's reason for dropout<br>*Field type*: Multiline Textfield<br>*Variable name*: Patient_dropout_reason<br>*Field required*: Not required	
44.2	Withdrawal from PI<br>*Field type*: Radiobutton<br>*Variable name*: PI_withdrew<br>*Field required*: Not required<br>*Option group name*: Yes/No	○ Yes<br>○ No
44.2.1	***If 'Withdrawal from PI' is equal to 'Yes' answer this question:***<br>PI's reason for withdrawal<br>*Field type*: Multiline Textfield<br>*Variable name*: PI_withdrawal_reason<br>*Field required*: Not required	

45. Any point during trial - Serious Adverse Event

Number	Question	Answers
45.1	Serious Adverse Event<br>*Field type*: Add report button<br>*Variable name*:<br>*Field required*: Not required	
